# *L1TD1* - a prognostic marker for colon cancer

**DOI:** 10.1186/s12885-019-5952-2

**Published:** 2019-07-23

**Authors:** Deepankar Chakroborty, Maheswara Reddy Emani, Riku Klén, Camilla Böckelman, Jaana Hagström, Caj Haglund, Ari Ristimäki, Riitta Lahesmaa, Laura L. Elo

**Affiliations:** 1 Turku Bioscience Centre, University of Turku and Åbo Akademi University, Turku, Finland; 2 Institute of Biomedicine, Faculty of Medicine, University of Turku, Turku, Finland; 3 Research Programs Unit, Translational Cancer Biology, University of Helsinki, Helsinki, Finland; 4 Department of Surgery, University of Helsinki and Helsinki University Hospital, Helsinki, Finland; 5 Department of Pathology and Oral Pathology, University of Helsinki and Helsinki University Hospital, Helsinki, Finland; 6 Department of Pathology, HUSLAB, Helsinki University Hospital and University of Helsinki, Helsinki, Finland; 7 Genome-Scale Biology Research program, University of Helsinki, 00290 Helsinki, Finland

**Keywords:** Human *L1TD1* gene, Colon cancer, Prognostic factors, Biomarkers, Survival analysis

## Abstract

**Background:**

Prognostic markers specific to a particular cancer type can assist in the evaluation of survival probability of patients and help clinicians to assess the available treatment modalities.

**Methods:**

Gene expression data was analyzed from three independent colon cancer microarray gene expression data sets (*N* = 1052). Survival analysis was performed for the three data sets, stratified by the expression level of the LINE-1 type transposase domain containing 1 (*L1TD1*). Correlation analysis was performed to investigate the role of the interactome of L1TD1 in colon cancer patients.

**Results:**

We found *L1TD1* as a novel positive prognostic marker for colon cancer. Increased expression of *L1TD1* associated with longer disease-free survival in all the three data sets. Our results were in contrast to a previous study on medulloblastoma, where high expression of *L1TD1* was linked with poor prognosis. Notably, in medulloblastoma *L1TD1* was co-expressed with its interaction partners, whereas our analysis revealed lack of co-expression of *L1TD1* with its interaction partners in colon cancer.

**Conclusions:**

Our results identify increased expression of *L1TD1* as a prognostic marker predicting longer disease-free survival in colon cancer patients.

**Electronic supplementary material:**

The online version of this article (10.1186/s12885-019-5952-2) contains supplementary material, which is available to authorized users.

## Background

Colon cancer is the third leading cancer, both in terms of newly diagnosed cases and mortality [1]. Despite the fact that chemotherapeutic agents, such as oxaliplatin and irinotecan, have markedly improved the survival rate in colon cancer [2], identification of patients likely to respond well to chemotherapy could increase the survival rate. Our study identifies LINE-1 type transposase domain containing 1 (*L1TD1*) as a novel positive prognostic marker for colon cancer.

Stem cell-like gene signatures have been detected in various cancers [3, 4], and embryonic stem cell factors have been associated with enhanced tumorigenesis and poor prognosis [5–7]. L1TD1 is an RNA-binding protein required for self-renewal of undifferentiated embryonic stem cells [8]. Recently, L1TD1 protein was shown to form a core interaction network with the canonical pluripotency factors OCT4, NANOG, LIN28, and SOX2 in human embryonic stem cells (hESCs) [9], and *L1TD1* depletion resulted in downregulation of the pluripotency markers *OCT4*, *NANOG*, and *LIN28* in hESCs [10]. L1TD1 has previously been shown to be essential for self-renewal of embryonal carcinoma cells [10] and to support the growth of seminoma cells [10].

We studied L1TD1 immunoexpression in colon adenocarcinoma tissue sections and analyzed three independent gene expression microarray data sets of colon cancer patients to assess the prognostic significance of *L1TD1* in colon cancer [11–13]. Our findings suggest that *L1TD1* is a promising prognostic marker for colon cancer.

## Methods

### Microarray data sets

Raw microarray data sets (Table 1) were downloaded from Gene Expression Omnibus (GEO) [17]. Three colon cancer gene expression microarray data sets comprising a total of 1052 clinical samples were analyzed [11–13]. Either due to non-tumoral origin (i.e. normal tissue) or due to missing associated survival information, 124 samples had to be excluded from the survival analysis (928 samples remained). Additionally, two seminoma [14, 15] and one stem cell [16] gene expression microarray data sets were analyzed to assess the co-expression of *L1TD1* and its interaction partners (Additional file 2: Table S1). The stem cell data set was composed of samples from ten hESCs, 49 induced pluripotent stem cells (iPSCs), five cancer cell lines, and six non-cancerous somatic cell lines. A summary of the data sets used is presented in Table 1.

**Table 1:** Summary of the data sets used in the study. The GEO accession numbers (GEO ID) are listed together with alias names used to refer to the individual data sets, the microarray platform, the total number of samples, and the number of samples used in the survival analysis

GEO ID	Total Samples	Survival Analysis	Platform	Alias
GSE14333 [11]	290	226	Affymetrix HG-U133Plus2	colon1
GSE17536 [12]	177	145	Affymetrix HG-U133Plus2	colon2
GSE39582 [13]	585	557	Affymetrix HG-U133Plus2	colon3
GSE3218 [14]	107	Not used	Affymetrix HG-U133A	seminoma1
GSE10783 [15]	34	Not used	Affymetrix HG-U133A	seminoma2
GSE42445 [16]	70	Not used	Agilent-028004 SurePrint G3 Human GE 8x60K	hESC1

### Gene expression analysis

The CEL files, containing the probe intensity measurements of the Affymetrix probes were normalized using the Universal exPression Code (UPC) normalization method from the Bioconductor package “SCAN.UPC” [18] and the Robust Multiarray Average (RMA) normalization method from the Bioconductor package “affy” [19, 20]. The UPC normalization method provides a score between 0.0 and 1.0, which represents the probability of a particular gene being expressed in a particular sample [18]. The UPC scores were used to categorize the samples in all data sets based on their *L1TD1* expression status as *L1TD1* high (UPC > =0.60) and *L1TD1* low (UPC < 0.60). The UPC threshold of 0.6 was determined by calculating a weighted mean (by sample size) of the local minima between the two peaks in the bimodal distributions of UPC scores for L1TD1 over the three colon cancer data sets (Additional file 1: Fig. S1). RMA provides normalized log_2_ intensity values. RMA normalized gene expression values were used to calculate pairwise correlations between genes. To correct for multiple testing, the false discovery rate (FDR) was controlled using the Benjamini-Hochberg procedure [21]. The probe “219955_at” was chosen as the primary probe for the quantification of *L1TD1* because it was present in both of the Affymetrix platforms used in this study (HG-U133Plus2 and HG-U133A).

### Gene list descriptions

#### Interaction partners

The 311 interaction partners of L1TD1 were determined using mass spectrometry and co-immunoprecipitation in our earlier study [9]. 306 interaction partners of L1TD1 were identified by performing a mass spectrometry analysis on co-immunoprecipitated proteins with two different anti-L1TD1 antibodies (recognizing different epitopes on L1TD1). In addition, for 5 proteins (NANOG, OCT4 (POU5F1), SOX2, DNMT3B, and TRIM28) that were challenging to detect using mass spectrometry, the interactions were shown using immunoprecipitation and Western blotting. Out of the 311 interaction partners, 285 corresponded to genes that had probes associated to them in the microarray platforms used in this study.

#### Top 20 interaction partners

The top 20 interaction partners of *L1TD1* were determined on the basis of their co-expression with *L1TD1* in the seminoma and stem cell data sets. First, the interaction partners were ranked in descending order of their Spearman rank correlation value with *L1TD1* in these data sets. Then, the maximum rank over the data sets was selected as a representative statistic for each interaction partner. The list was ordered (ascending) based on this maximum rank and 20 interaction partners were selected from the top of the list.

#### Top 20 co-expressed genes with *L1TD1* in colon cancer

Out of all the genes in the microarray data sets (27213 unique probe-gene mappings), top 20 genes were selected based on their co-expression with *L1TD1* in the colon cancer data sets. First, all the genes in the microarray data sets were ranked in descending order of their Spearman rank correlation value with *L1TD1* separately for each colon cancer data set. Then, the maximum rank over these data sets was selected as a representative statistic for each gene. The list was ordered (ascending) based on this maximum rank and 20 genes were selected from the top of the list.

### Survival analysis of microarray data

Disease-free survival was analyzed in each data set with the Kaplan-Meier method as implemented in the R package “survival” [22, 23] and survival curves were plotted using the R package “survminer” [24]. The log-rank test was used to compare survival rates between the two *L1TD1* groups (*L1TD1* high and *L1TD1* low).

### Association between *L1TD1* expression and clinicopathological variables

We investigated the association of age and sex and other publicly accessible clinicopathological variables to the *L1TD1* gene expression in the three gene expression data sets. The variables included cancer stage [11–13], prior-therapy received by the patients [11–13], tumor location [11–13], chromosomal instability [13], CpG island methylation status [13], DNA mismatch repair proficiency [13], mutation status of BRAF (B-Raf proto-oncogene, serine/threonine kinase), mutation status of KRAS (KRAS proto-oncogene, GTPase), and mutation status of TP53 (Tumor Protein p53) [13]. For variables with only two categories, Wilcoxon rank sum test [25] was used for the analysis of statistical significance. For variables with more than two categories, Kruskal-Wallis test [26] was used. Association of *L1TD1* expression with age was investigated using Pearson correlation [27].

### Analysis of TCGA Colon adenocarcinoma RNA-seq data set

RNA-seq data from The Cancer Genome Atlas Colon Adenocarcinoma [28] (TCGA-COAD) data set was acquired from Genomic Data Commons (portal.gdc.cancer.gov). The FPKM-UQ normalized (Fragments Per Kilobase of transcript per Million mapped reads Upper Quartile) RNA-seq counts from the primary tumor samples (*N* = 521) were used to validate the correlation analyses performed using the microarray data sets. Due to lack of an evident choice of the intensity threshold to designate samples into high and low *L1TD1* expression groups, we fitted a mixture of two Gaussian distributions and evaluated two different thresholds (Additional file 1: Figure S2): FPKM-UQ value where the ratio of the two Gaussian distributions was equal, and FPKM-UQ value where the ratio of the two Gaussian distributions was 10%. These two thresholds were then used to perform survival analysis using disease-free survival with Kaplan-Meier method.

## Results

### High expression of *L1TD1* associates with longer disease-free survival

Across the three colon cancer microarray data sets, 26.7% of the colon cancer patients were categorized to have high *L1TD1* expression (Table 2, Additional file 1: Figure S3). The proportion was lower than that observed in seminoma (48.6 and 50%) and stem cell (88.6%) data sets (Table 2, Additional file 1: Figure S3).

**Table 2:** Proportion of samples with high expression of *L1TD1*. The samples were categorized based on their *L1TD1* expression level (high *L1TD1*+ or low *L1TD1*-) in the different data sets used in this study. For the colon cancer data sets, only tumor samples with complete survival information were considered

Data set	*L1TD1* +	*L1TD1* -	Total	Percentage of *L1TD1* +
colon1	64	162	226	28.3%
colon2	44	101	145	30.3%
colon3	140	417	557	25.1%
Total (Colon Cancer)	248	680	928	26.7%
seminoma1	52	55	107	48.6%
seminoma2	17	17	34	50.0%
hESC1	62	8	70	88.6%

**Fig. 1:**
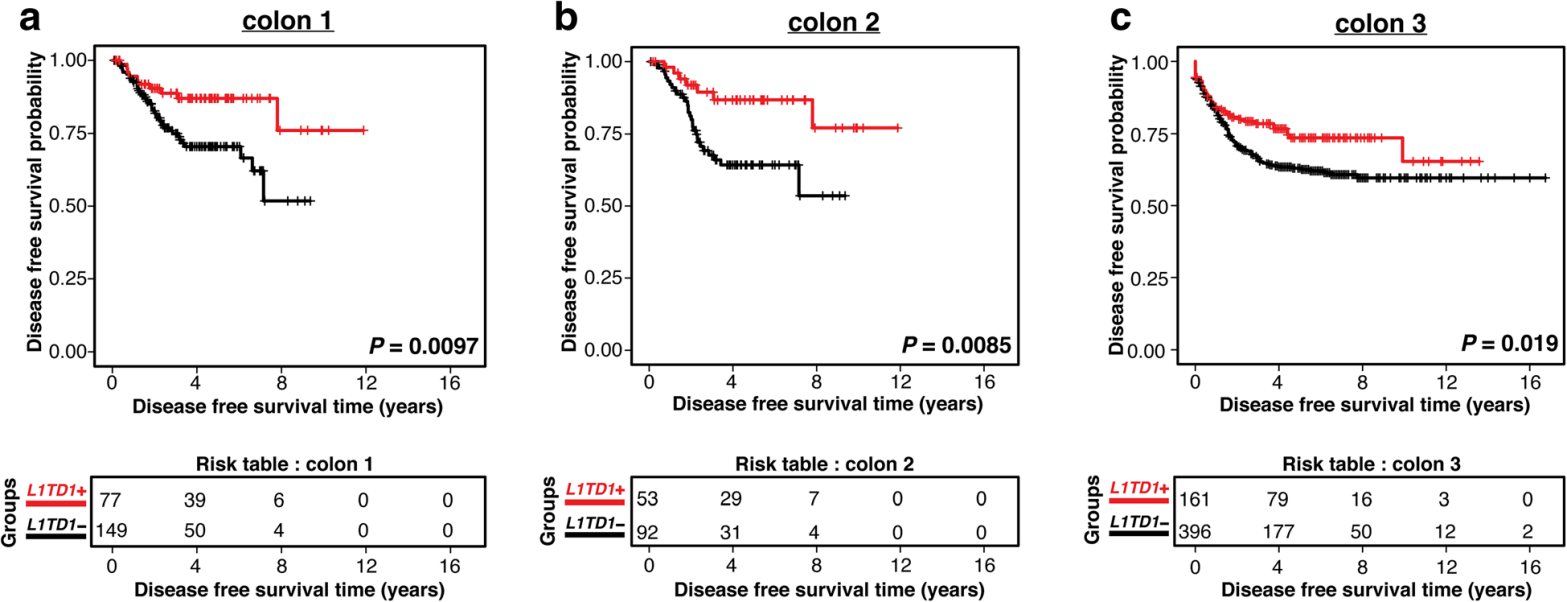
Survival curves for colon cancer. Kaplan-Meier curves showing disease-free survival for the three colon cancer data sets (**a**-**c**). The curves present survival data for the two groups of colon cancer patients based on *L1TD1* expression level (high or low). The red curve corresponds to the patients with high *L1TD1* expression and the black curve corresponds to the patients with low *L1TD1* expression. The *x*-axis shows disease-free survival time in years and the *y*-axis shows the probability of disease-free survival. The risk table shows the number of patients at risk at the given time point

Kaplan-Meier analysis of 928 samples from the three colon cancer data sets revealed that the colon cancer samples with high *L1TD1* expression had longer disease-free survival as compared to those with no/low *L1TD1* expression (Fig. 1). The difference was statistically significant in all the three data sets (log-rank test *P* < 0.05).

*L1TD1* expression was higher in the samples from early cancer stages as compared to those from later stages in all the three data sets (*P* < 0.05), whereas differences between the later stages were typically not statistically significant (Additional file 1: Figure S4A-C). In the dataset colon3, *L1TD1* expression was high for samples with mutated KRAS (*P* < 0.0001), wild-type TP53 (*P* < 0.0001), and negative chromosomal instability marker (*P* < 0.0001) (Additional file 1: Figure S4D-F). Additionally, significant associations were observed between *L1TD1* expression and tumor location or tumor differentiation status (*P* < 0.0001) (Additional file 1: Figure S4G-I). Age, sex, prior therapy (chemo-, radio- or adjuvant therapy), BRAF mutation status, CpG island methylation status, or DNA mismatch repair proficiency did not show statistically significant associations with the *L1TD1* expression (Additional file 1: Figure S5).

### Interactome of *L1TD1* is not co-expressed in colon cancer

**Fig. 2:**
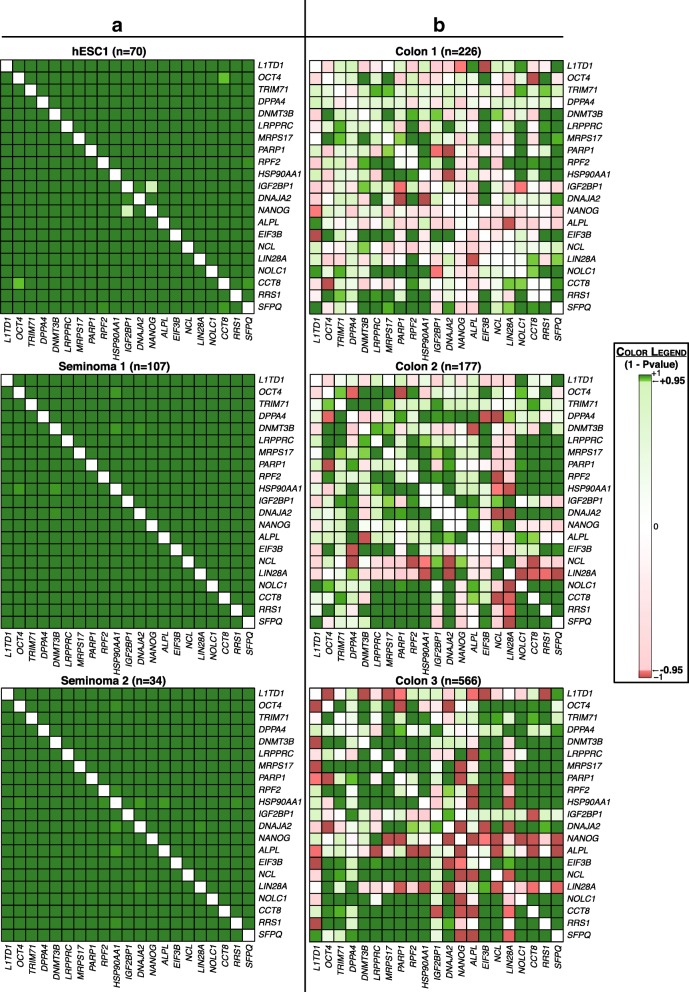
Co-expression of interaction partners of *L1TD1***.** Heatmaps showing signed *P*-value of Spearman rank correlation for the 20 most significantly co-expressed interaction partners of *L1TD1* determined on the basis of the seminoma and stem cell data sets. Co-expression in (**a**) seminoma and stem cell data sets, and (**b**) colon cancer data sets. The signed *P*-value of Spearman rank correlation was defined as 1 - *P*-value of Spearman rank correlation multiplied by the sign of the correlation

To examine the potential role of the previously identified interaction partners of *L1TD1* [9] (Additional file 2: Table S1) in its prognostic performance in colon cancer, Spearman rank correlation matrices were calculated between the expression levels of *L1TD1* and its interaction partners [9]. Interestingly, the high positive correlation observed among *L1TD1* and its top 20 interaction partners in seminoma and stem cell data sets (P < 0.0001, Fig. 2a) was absent in all three colon cancer data sets (Fig. 2b). However, the interaction partners did not consistently improve the predictive prognostic power obtained with *L1TD1* alone (Additional file 2: Table S2).

### Genes co-expressed with *L1TD1* in colon cancer

We identified genes that were co-expressed with *L1TD1* in colon cancer patients using Spearman rank correlation (Table 3, Additional file 2: Table S3). Although none of the top 20 co-expressed genes outperformed *L1TD1* as independent prognostic marker for colon cancer in all the three data sets, five genes had statistically significant (*P* < 0.05) impact on survival in at least two out of the three colon cancer data sets (Table 4): Serine peptidase inhibitor Kazal type 4 (*SPINK4*), Resistin-like beta (*RETNLB*), Asparaginase-like 1 Protein (*ASRGL1*), Chloride channel accessory 1 (*CLCA1*), and Fc fragment of IgG binding protein (*FCGBP*) (Additional file 1: Figure S6).

**Table 3:** Top 20 co-expressed genes with *L1TD1* in colon cancer. The Spearman rank correlation values (*r*_*s*_) with *L1TD1* are shown together with their false discovery rates (FDR) separately for each colon cancer data set

		colon 1		colon 2		colon 3	
Rank	Gene Name	*r*_*s*_	FDR	*r*_*s*_	FDR	*r*_*s*_	FDR
1	*RETNLB*	0.47	9.26E-13	0.53	3.69E-10	0.45	0.00
2	*CLCA1*	0.45	5.65E-12	0.43	1.10E-05	0.45	0.00
3	*HEPACAM2*	0.43	1.05E-10	0.41	3.98E-05	0.46	0.00
4	*FOXA3*	0.41	1.14E-09	0.43	1.06E-05	0.43	0.00
5	*FCGBP*	0.41	1.14E-09	0.39	2.15E-04	0.47	0.00
6	*ST6GALNAC1*	0.40	4.55E-09	0.39	1.87E-04	0.43	2.57E-24
7	*SPINK4*	0.44	2.99E-11	0.38	3.91E-04	0.43	5.06E-25
8	*KIAA1324*	0.40	4.60E-09	0.44	7.71E-06	0.39	0.00
9	*KLF4*	0.40	4.60E-09	0.37	4.61E-04	0.41	0.00
10	*GMDS*	0.46	1.50E-12	0.40	9.95E-05	0.38	0.00
11	*SLITRK6*	0.43	5.87E-11	0.36	1.14E-03	0.46	0.00
12	*SERPINA1*	0.42	1.35E-10	0.38	3.84E-04	0.35	1.26E-16
13	*LINC00261*	0.34	1.45E-06	0.35	2.09E-03	0.48	0.00
14	*ITLN1*	0.35	4.43E-07	0.33	3.97E-03	0.42	0.00
15	*MUC2*	0.39	8.64E-09	0.33	4.90E-03	0.38	0.00
16	*DEFA5*	0.37	5.72E-08	0.35	1.78E-03	0.33	6.77E-14
17	*ASRGL1*	0.40	4.55E-09	0.32	6.22E-03	0.41	0.00
18	*SLC27A2*	0.36	2.17E-07	0.36	9.05E-04	0.33	2.44E-13
19	*RNF186*	0.32	8.44E-06	0.36	1.30E-03	0.34	1.89E-14
20	*PCCA*	0.37	1.05E-07	0.37	7.52E-04	0.33	2.95E-13

**Table 4:** Prognostic assessment of genes that co-express with *L1TD1* in colon cancer

Gene	colon1	colon2	colon3
*L1TD1*	0.009729	0.008520	0.018607
*SPINK4*	0.007148	0.001854	0.880992
*RETNLB*	0.325642	0.012519	0.009064
*ASRGL1*	0.015986	0.521116	0.016293
*CLCA1*	0.030053	0.006496	0.710961
*FCGBP*	0.028617	0.047080	0.292182
*ITLN1*	0.088225	0.043802	0.844453
*FOXA3*	0.077752	0.609721	0.093598
*PCCA*	0.064797	0.601176	0.107992
*DEFA5*	0.136904	0.157008	0.737800
*GMDS*	0.318171	0.170255	0.000919
*HEPACAM2*	0.368837	0.687066	0.098125
*SERPINA1*	0.000008	0.493419	0.911649
*RNF186*	0.700045	0.541107	0.010793
*KLF4*	0.938136	NA	0.220231
*ST6GALNAC1*	0.593332	0.880638	0.030027
*MUC2*	0.624983	0.505661	0.842770
*KIAA1324*	0.220079	0.969530	0.730810
*SLITRK6*	0.750696	0.894483	0.085490
*LINC00261*	0.823520	0.823442	0.269044
*SLC27A2*	0.883481	0.975288	0.002906

Statistical significance of the top 20 co-expressed genes in the survival analysis of colon cancer patients in the three data sets. Genes with statistically significant association with disease-free survival (log-rank test *P* < 0.05) in at least two colon cancer data sets are underlined

### Validation in TCGA Colon adenocarcinoma RNA-seq data set

To further validate our findings from the three colon cancer microarray data sets, we analyzed the TCGA Colon Adenocarcinoma [28] (TCGA-COAD) RNA-seq data set containing 521 patient samples. When the samples were stratified for *L1TD1* expression using the threshold where the ratio of the two Gaussian distributions was 10%, Kaplan-Meier analysis supported that the colon cancer samples with high *L1TD1* expression had longer disease-free survival as compared to those with no/low *L1TD1* expression (*P* = 0.038, Additional file 1: Figure S2C). Additionally, we were able to reproduce the findings from the correlation analyses, indicating a lack of correlation between *L1TD1* and its top 20 interaction partners (Additional file 1: Figure S2D) and confirming significant correlations between *L1TD1* and genes that were co-expressed with *L1TD1* in the colon cancer microarray data sets (Additional file 1: Figure S2E).

## Discussion

In this study, we examined the prognostic value of *L1TD1* in colon cancer patients. We found compelling evidence of *L1TD1* being a positive prognostic marker for colon cancer (Fig. 1). We demonstrated this by survival analysis of 928 samples from three independent gene expression data sets of colon cancer patients and further confirmed the results in the TCGA Colon Adenocarcinoma RNA-seq data set of 521 colon cancer patients.

Expression of *L1TD1* has earlier been reported to be highly specific to embryonic stem cells [10], brain [29], and colon (Additional file 1: Figure S7). Besides these healthy tissues, *L1TD1* expression has also been reported in seminoma [10], embryonic carcinomas [10], medulloblastoma [30], and colon adenocarcinoma (Additional file 1: Figures S3 and S7). Expression of *L1TD1* at high levels in colon cancer cells led us to hypothesize that high expression of *L1TD1* in colon cancer might be associated with prognosis. Earlier reports have demonstrated the association of stem cell pluripotency factors with poor prognosis in different cancer types, including medulloblastoma [30] and seminoma [15]. Interestingly, our results were in contrast with previous studies, suggesting that in colon cancer, high expression of *L1TD1* is linked to better prognosis. In the three colon cancer data sets, expression of L1TD1 was associated with samples of low clinical cancer stage (Additional file 1: Figure S4A-C), which can perhaps be a reason for its prognostic significance.

In an attempt to understand the distinctive role of *L1TD1* in different cancers, we investigated the co-expression of *L1TD1* with its currently known interaction partners. We discovered that, unlike in hESCs and seminomas, *L1TD1* was not co-expressed with its interaction partners in colon cancer (Fig. 2). This points to the potential participation of *L1TD1*’s interaction partners in the contrasting prognostic outcome. This was further supported by a recent study in medulloblastoma, showing an association of high *L1TD1* expression with poor clinical outcome and significant co-expression between *L1TD1* and its interaction partner *OCT4* [30]. Together, these findings suggest that the co-expression of *L1TD1* with its interaction partners might be required for manifesting an aggressive and detrimental phenotype. This is the first time that an embryonic stem cell factor has been shown to lead to contrasting outcomes in cancer, taking into consideration to the presence or absence of strong co-expression with its interaction partners.

We also investigated genes that were co-expressed with *L1TD1* in colon cancer. Among the top 20 co-expressed genes, six had previously been linked to colon cancer. Chloride Channel Accessory 1 (*CLCA1*) is a tumor suppressor protein that regulates differentiation and proliferation of colorectal cancer cells. Its low expression has been associated with tumorigenesis, metastasis, and chromosomal instability, as well as poor prognosis in colorectal cancer [31]. Kruppel Like Factor 4 (*KLF4*) is a target of the tumor suppressor gene Adenomatous Polyposis Coli (*APC*) and its overexpression reduces cell migration and invasion in vitro and tumorigenicity of colon cancer cells in vivo [32]. GDP-mannose-4,6-dehydratase (*GMDS*) has been shown to have exon deletions linked to progression of colorectal cancer [33]. Also, an in vitro study found that GMDS deficiency in colon cancer cells made them resistant to receptor-mediated apoptosis [34]. High expression of Mucin 2 (*MUC2*) has been associated with longer disease-free survival in colorectal cancer patients [35]. Frameshift mutations resulting in premature termination of translation of Propionyl-CoA Carboxylase Alpha Subunit (*PCCA*) have been reported in colon and gastric cancer [36]. Investigation of the potential role of Alpha-1-antitrypsin (*SERPINA1*) expression in cancers provides controversial results; it has been associated with good prognosis in breast and colon cancer on protein atlas [37] (https://www.proteinatlas.org/ENSG00000197249-SERPINA1/pathology), but there are also reports that associate it with poor prognosis in colon cancer [38], gastric cancer [39] and cutaneous squamous cell carcinoma [40].

Several of the co-expressed genes have been linked to various other cancers. Down-regulation of Fc fragment of IgG binding protein (*FCGBP*) has been associated with decreased overall survival in gallbladder adenocarcinoma [41] and with progression of prostate cancer in Transgenic adenocarcinoma Mouse Prostate (*TRAMP*) [42]. Upregulation of ST6 N-acetylgalactosaminide alpha-2,6-sialyltransferase 1 (*ST6GALNAC1*) has been associated with good prognosis in breast cancer [43]. Additionally, siRNA-mediated silencing of *ST6GALNAC1* has been shown to lead to reduced growth, migration and invasion of gastric cancer cells in vitro [44]. Estrogen-Induced Gene 121 Protein (*KIAA1324*), Long Intergenic Non-Protein Coding RNA 261 (*LINC00261*), and Intelectin 1 (ITLN1) have been shown to function as tumor suppressors in gastric cancer, with decreased expression associated with poor prognosis [45–47]. Low expression of Asparaginase-Like 1 Protein (*ASRGL1*) has been suggested as a marker for poor prognosis in endometrial carcinoma [48], whereas reduced levels of Solute carrier family 27 member 2 (*SLC27A2*) have been associated with poor survival in lung cancer [49]. SLIT and NTRK- like protein 6 (*SLITRK6*) is a known bladder tumor antigen, and is currently under investigation in clinical trials as a target for antibody-drug conjugate therapy [50]. *HEPACAM* family member 2 (*HEPACAM2*) is a paralog of Hepatocyte Cell Adhesion Molecule (*HEPACAM*), which is known to act as a tumor suppressor by promoting differentiation [51]. *HEPACAM2*, however, is a relatively newly-identified molecule and is not well-studied.

## Conclusion

Our study of gene expression data from four clinical colon cancer data sets produced promising evidence in support of *L1TD1* as a marker for good prognosis in colon cancer. Our results emphasize the need for further investigation and validation of *L1TD1* as a potential prognostic marker in larger cohorts of colon cancer. Finally, this work also underscores the potential merits of investigating co-expressed genes to markers of interest.

## Additional files

Additional file 1: **Figure S1.** Density distributions of UPC scores for L1TD1 in the three colon cancer microarray data sets. A dashed black line indicates the UPC threshold of 0.6, which was used to stratify the samples into L1TD1+ and L1TD1- groups in the three data sets. **Figure S2.** Analysis of the primary tumor samples in The Cancer Genome Atlas Colon Adenocarcinoma (TCGA-COAD) data set. (**A**) Estimation of FPKM-UQ normalized RNA-seq counts by fitting Gaussian distributions. (**B-C**) Kaplan-Meier curves using the two thresholds for designating L1TD1 high and low samples (grey and red dashed lines, respectively). (**D**) Heatmaps showing signed P-value of Spearman rank correlation for the 20 most significantly co-expressed interaction partners of L1TD1. (**E**) The Spearman rank correlation values (rs) between L1TD1 and its top 20 co-expressed genes (Table 3). The correlations in the TCGA-COAD data set are shown with their false discovery rate (FDR). **Figure S3.** Heatmaps showing expression level of L1TD1 and its top 20 interaction partners in the samples of (**A**) colon cancer data sets, and (**B**) seminoma and stem cell data sets. **Figure S4.** Boxplots of UPC scores of L1TD1 stratified based on the indicated clinicopathological parameters in the different colon cancer microarray data sets. **Figure S5.** Boxplots of UPC scores of L1TD1 stratified based on the indicated clinicopathological parameters in the different colon cancer microarray data sets. **Figure S6.** Kaplan-Meier curves showing disease-free survival for the three colon cancer data sets (columns). The curves present survival data for the two groups of colon cancer patients based on gene expression level (high or low) of SPINK4, RETNLB, ASRGL1, CLCA1, and FCGBP (rows). Grey = high gene expression, Black = low gene expression. **Figure S7.** Formalin-fixed and paraffin-embedded tissue microarray blocks were stained with immunohistochemistry using anti-L1TD1 (Atlas Antibodies, HPA028501). (**A**) Normal colon tissue, (**B**) colorectal adenocarcinoma sample. (PDF 2132 kb)

Additional file 2: **Table S1.** 311 Interaction partners of L1TD1 were determined using Mass spectrometry and co-immunoprecipitation in our earlier publication (Emani, Närvä, 2015, Stem cell reports). 306 Interaction partners of L1TD1 were identified by performing a Mass spectrometry analysis on co-immunoprecipitated proteins with two different anti-L1TD1 antibodies (recognizing different epitopes on L1TD1). In addition, we included 5 more proteins (NANOG, OCT4 (POU5F1), SOX2, DNMT3B, and TRIM28) that were challenging to detect using Mass spectrometry but the interactions were shown using Immunoprecipitation and Western Blotting. This makes a total of 311 proteins that are referred to in this work as “Interaction partners” of L1TD1. **Table S2.** Colon cancer samples with a high L1TD1 expression and a concomitant lack of expression of the listed interaction partner were compared to colon cancer samples with a low L1TD1 expression in the three data sets, this table lists the *P*-values (log-rank test) for these comparisons. *P*-value less (more significant) than the one obtained by comparing L1TD1 high and low sample groups are highlighted. **Table S3.** Table lists the 20 genes that had a positive correlation with L1TD1 in the colon cancer data sets. The table lists their UNIPROT ID and UNIRPOT protein name. (PDF 211 kb)

## Data Availability

All requests for access to data and material are to be addressed jointly to Riitta Lahesmaa and Laura L. Elo. Publicly available datasets can be accessed at Gene Expression Omnibus (GEO IDs listed in Table 1).

## Abbreviations

APC: Adenomatous Polyposis Coli
ASRGL1: Asparaginase-like 1 Protein
CLCA1: Chloride channel accessory 1
DNMT3B: DNA (cytosine-5)-methyltransferase 3B
FCGBP: Fc fragment of IgG binding protein
FDR: False discovery rate
GEO: Gene Expression Omnibus
GMDS: GDP-mannose-4,6-dehydratase
HEPACAM: Hepatocyte Cell Adhesion Molecule
HEPACAM2: HEPACAM family member 2
hESC: Human embryonic stem cell
iPSCs: Induced pluripotent stem cell
ITLN1: Intelectin 1
KIAA1324: Estrogen-Induced Gene 121 Protein
KLF4: Kruppel Like Factor 4
L1TD1: LINE-1 type transposase domain containing 1
LIN28: Protein lin-28 homolog A
LINC00261: Long Intergenic Non-Protein Coding RNA 261
MUC2: Mucin 2
NANOG: Homeobox protein NANOG
OCT4: POU domain, class 5, transcription factor 1
PCCA: Propionyl-CoA Carboxylase Alpha Subunit
RETNLB: Resistin-like beta
RMA: Robust Multiarray Average
SERPINA1: Alpha-1-antitrypsin
SLC27A2: Solute carrier family 27 member 2
SLITRK6: SLIT and NTRK- like protein 6
SOX2: Transcription factor SOX-2
SPINK4: Serine peptidase inhibitor Kazal type 4
ST6GALNAC1: ST6 N-acetylgalactosaminide alpha-2,6-sialyltransferase 1
TRAMP: Transgenic adenocarcinoma Mouse Prostate
TRIM28: Transcription intermediary factor 1-beta
UPC: Universal exPression Code
