# Supplementary material for: L1TD1 - a prognostic marker for colon cancer
Source: BMC Cancer. 2019 Jul 23;19:727. doi: 10.1186/s12885-019-5952-2 (PMC6651905; doi:10.1186/s12885-019-5952-2)
Supplement: Supplementary file 1 — Figure S1. Density distributions of UPC scores for L1TD1 in the three colon cancer microarray data sets. A dashed black line indicates the UPC threshold of 0.6, which was used to stratify the samples into L1TD1+ and L1TD1- groups in the three data sets. Figure S2. Analysis of the primary tumor samples in The Cancer Genome Atlas Colon Adenocarcinoma (TCGA-COAD) data set. (A) Estimation of FPKM-UQ normalized RNA-seq counts by fitting Gaussian distributions. (B-C) Kaplan-Meier curves using the two thresholds for designating L1TD1 high and low samples (grey and red dashed lines, respectively). (D) Heatmaps showing signed P-value of Spearman rank correlation for the 20 most significantly co-expressed interaction partners of L1TD1. (E) The Spearman rank correlation values (rs) between L1TD1 and its top 20 co-expressed genes (Table 3). The correlations in the TCGA-COAD data set are shown with their false discovery rate (FDR). Figure S3. Heatmaps showing expression level of L1TD1 and its top 20 interaction partners in the samples of (A) colon cancer data sets, and (B) seminoma and stem cell data sets. Figure S4. Boxplots of UPC scores of L1TD1 stratified based on the indicated clinicopathological parameters in the different colon cancer microarray data sets. Figure S5. Boxplots of UPC scores of L1TD1 stratified based on the indicated clinicopathological parameters in the different colon cancer microarray data sets. Figure S6. Kaplan-Meier curves showing disease-free survival for the three colon cancer data sets (columns). The curves present survival data for the two groups of colon cancer patients based on gene expression level (high or low) of SPINK4, RETNLB, ASRGL1, CLCA1, and FCGBP (rows). Grey = high gene expression, Black = low gene expression. Figure S7. Formalin-fixed and paraffin-embedded tissue microarray blocks were stained with immunohistochemistry using anti-L1TD1 (Atlas Antibodies, HPA028501). (A) Normal colon tissue, (B) colorectal adenocarcinoma sample. (PDF [file 12885_2019_5952_MOESM1_ESM.pdf]

SI Figure 1

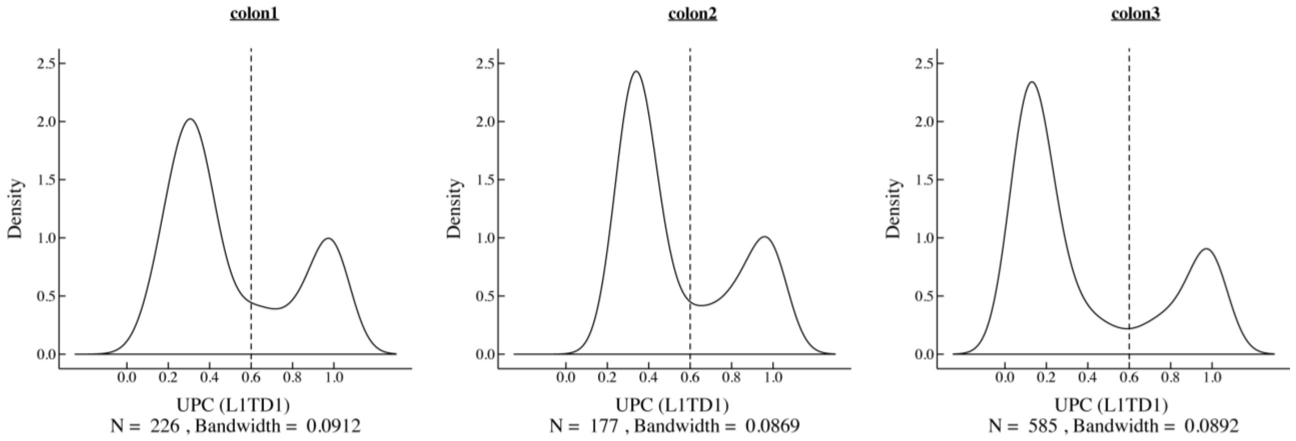

Supplementary Figure 1. Density distributions of UPC scores for L1TD1 in the three colon cancer microarray data sets used. A dashed black line indicates the UPC threshold of 0.6, which was used to stratify the samples into L1TD1+ and L1TD1- groups in the three data sets. The threshold was obtained by calculating a weighted mean of the minima between the two peaks in the bimodal distributions.

SI Figure 2

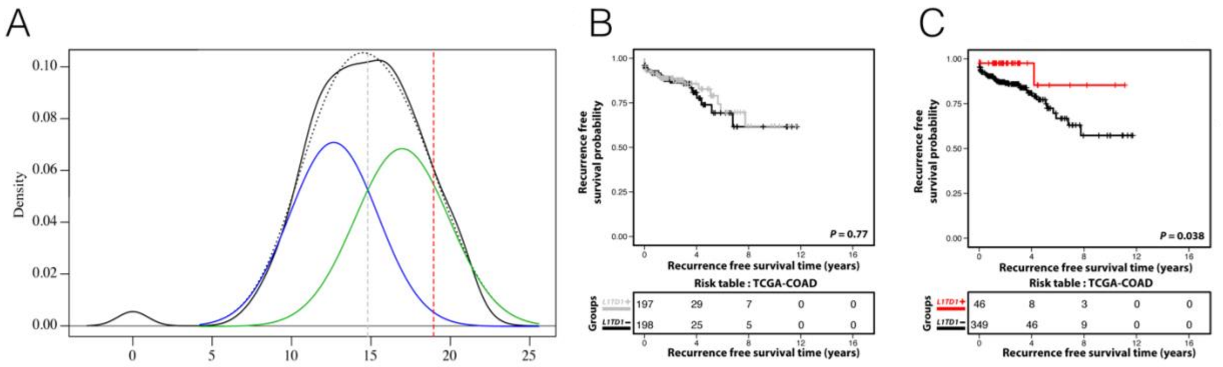

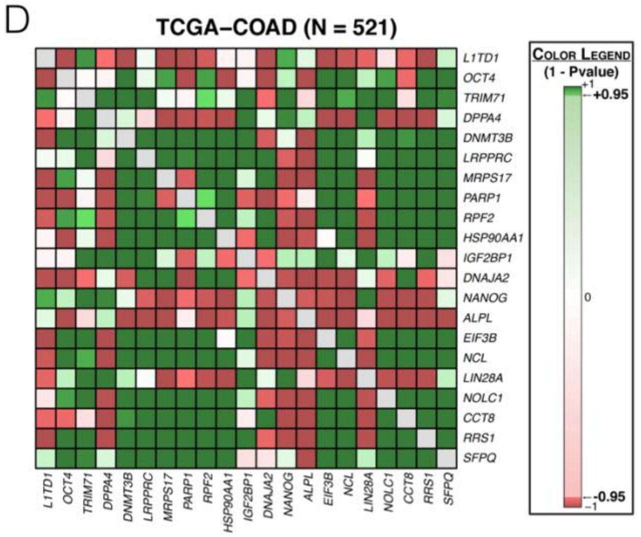

E

	TCGA-COAD	
Gene	r_s	FDR
RETNLB	0.52	4.47E-33
CLCA1	0.50	1.46E-30
HEPACAM2	0.51	1.98E-32
FOXA3	0.36	7.54E-15
FCGBP	0.48	1.90E-27
ST6GALNAC1	0.45	3.21E-24
SPINK4	0.41	6.47E-19
KIAA1324	0.45	1.37E-23
KLF4	0.38	1.78E-16
GMDS	0.40	4.71E-18
SLITRK6	0.53	1.60E-34
SERPINA1	0.28	9.02E-09
LINC00261	0.36	8.94E-15
ITLN1	0.37	6.88E-15
MUC2	0.39	2.00E-17
DEFA5	0.47	4.70E-27
ASRGL1	0.30	1.63E-09
SLC27A2	0.31	2.93E-10
RNF186	0.34	1.53E-12
PCCA	0.32	1.43E-11

Supplementary Figure 2. Analysis of the primary tumor samples in The Cancer Genome Atlas Colon Adenocarcinoma (TCGA-COAD) data set. **(A)** Estimation of the Fragments Per Kilobase of transcript per Million mapped reads upper quartile (FPKM-UQ) normalized RNA-seq counts (black solid curve) by fitting a mixture of two Gaussian distributions (green and blue curves). Two different thresholds were considered to designate the samples into high and low L1TD1 expression groups, including FPKM-UQ value where the ratio of the two Gaussian distributions was equal (vertical grey dashed line) and FPKM-UQ value where the ratio of the two Gaussian distributions was 10% (vertical red dashed line). **(B-C)** Kaplan-Meier curves using the two thresholds for designating L1TD1 high and low samples (grey and red dashed lines, respectively). The *x*-axis shows disease-free survival time in years and the *y*-axis shows the probability of disease-free survival. The risk table shows the number of patients at risk at the given time point. **(D)** Heatmaps showing signed P-value of Spearman rank correlation for the 20 most significantly co-expressed interaction partners of L1TD1 determined on the basis of the seminoma and stem cell data sets. The signed P-value of Spearman rank correlation was defined as 1 - P-value of Spearman rank correlation multiplied by the sign of the correlation. **(E)** The Spearman rank correlation values (rs) between L1TD1 and its top 20 co-expressed genes (see Table 3). The correlations in the TCGA-COAD data set are shown together with their false discovery rate (FDR).

SI Figure 3A

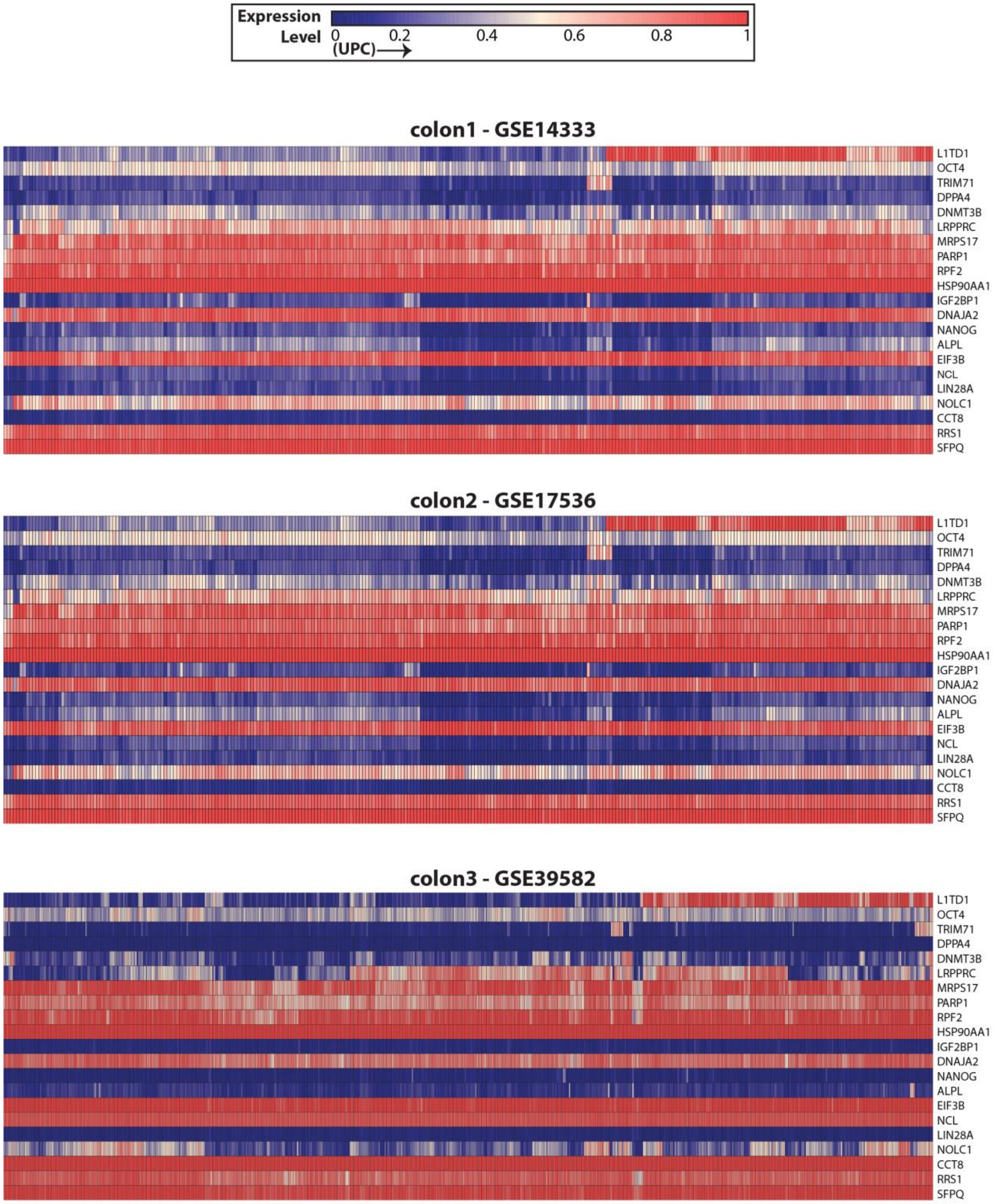
Expression
Level
0
0.2
0.4
0.6
0.8
1
(UPC)
colon1 - GSE14333
L1TD1
OCT4
TRIM71
DPPA4
DNMT3B
LRPPRC
MRPS17
PARP1
RPF2
HSP90AA1
IGF2BP1
DNAJA2
NANOG
ALPL
EIF3B
NCL
LIN28A
NOLC1
CCT8
RRS1
SFPQ
colon2 - GSE17536
L1TD1
OCT4
TRIM71
DPPA4
DNMT3B
LRPPRC
MRPS17
PARP1
RPF2
HSP90AA1
IGF2BP1
DNAJA2
NANOG
ALPL
EIF3B
NCL
LIN28A
NOLC1
CCT8
RRS1
SFPQ
colon3 - GSE39582
L1TD1
OCT4
TRIM71
DPPA4
DNMT3B
LRPPRC
MRPS17
PARP1
RPF2
HSP90AA1
IGF2BP1
DNAJA2
NANOG
ALPL
EIF3B
NCL
LIN28A
NOLC1
CCT8
RRS1
SFPQ

SI Figure 3B

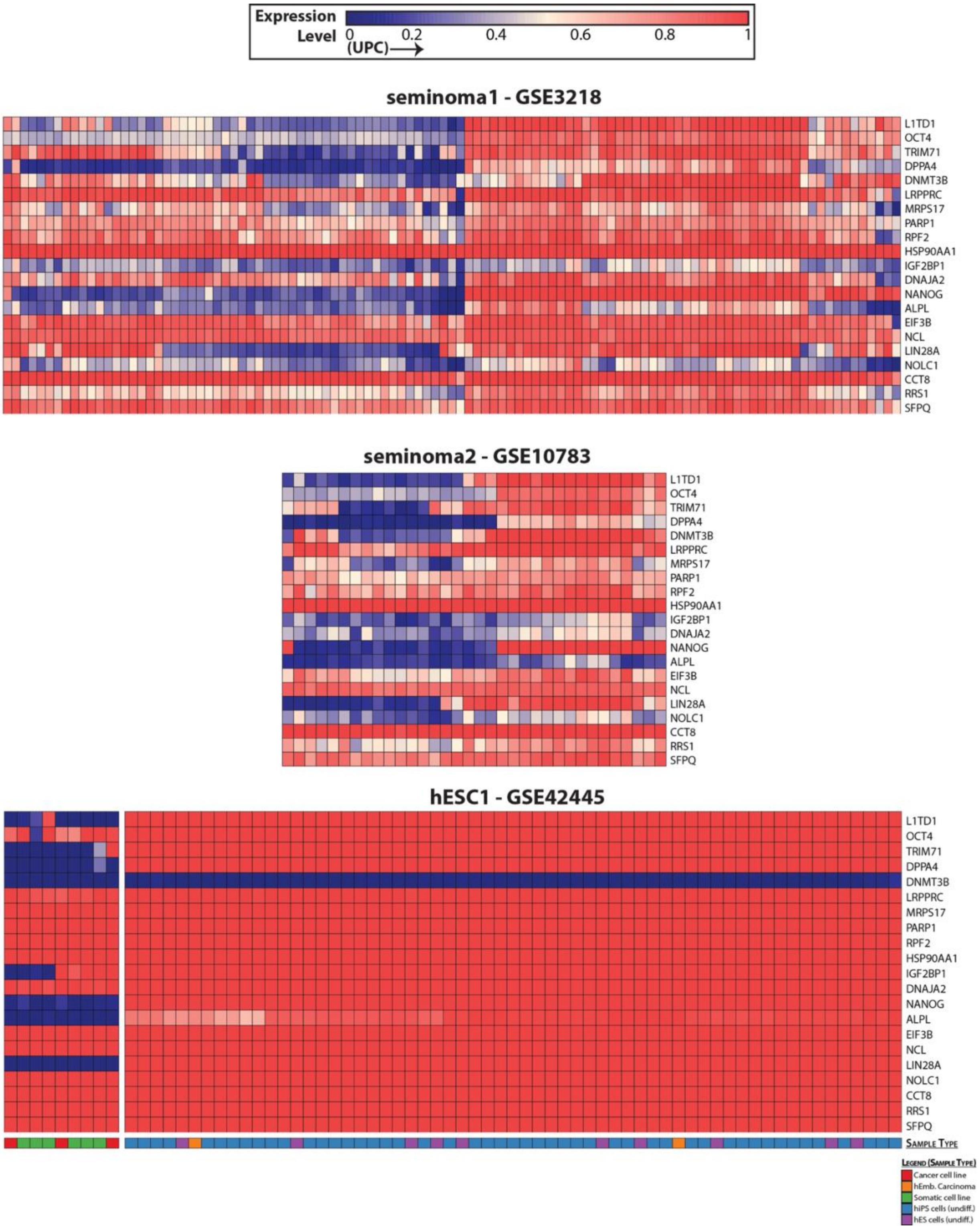

Supplementary Figure 3. Heatmaps showing expression level of L1TD1 and its top 20 interaction partners in the samples of **(A)** colon cancer data sets, and **(B)** seminoma and stem cell data sets used in this study. The samples (columns) in the heatmap are clustered based on Euclidean distance and the genes (rows) are ordered based on the order of interaction partners as in Supplementary Table 2.

SI Figure 4

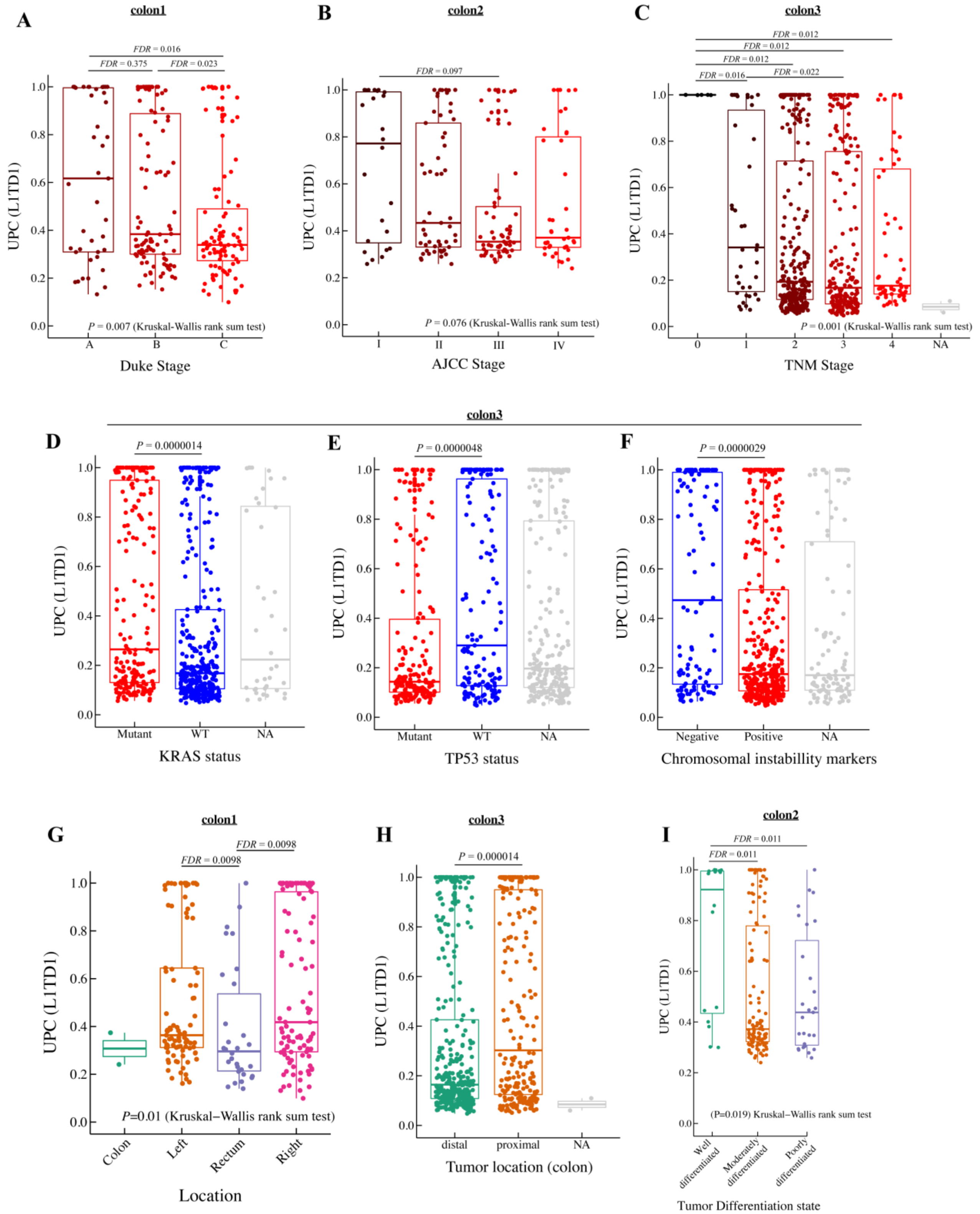

Supplementary Figure 4. Boxplots of UPC scores of L1TD1 stratified based on the indicated clinicopathological parameters in the different colon cancer microarray data sets. The significant pairwise differences are indicated by the horizontal lines above the groups being compared.

SI Figure 5

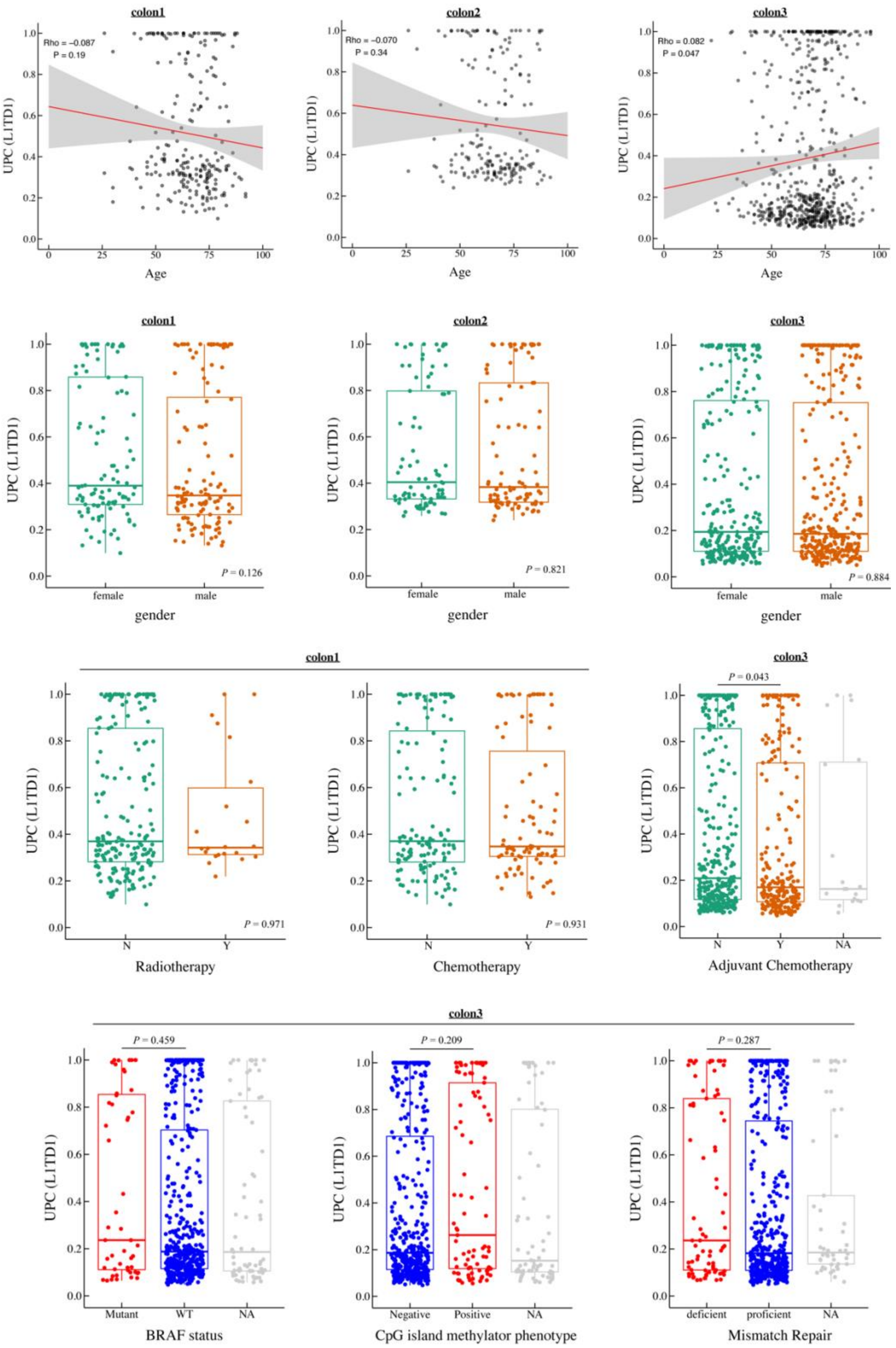

Supplementary Figure 5. Boxplots of UPC scores of L1TD1 stratified based on the indicated clinicopathological parameters in the different colon cancer microarray data sets. None of the comparisons were statistically significant.

Supplementary Figure 6

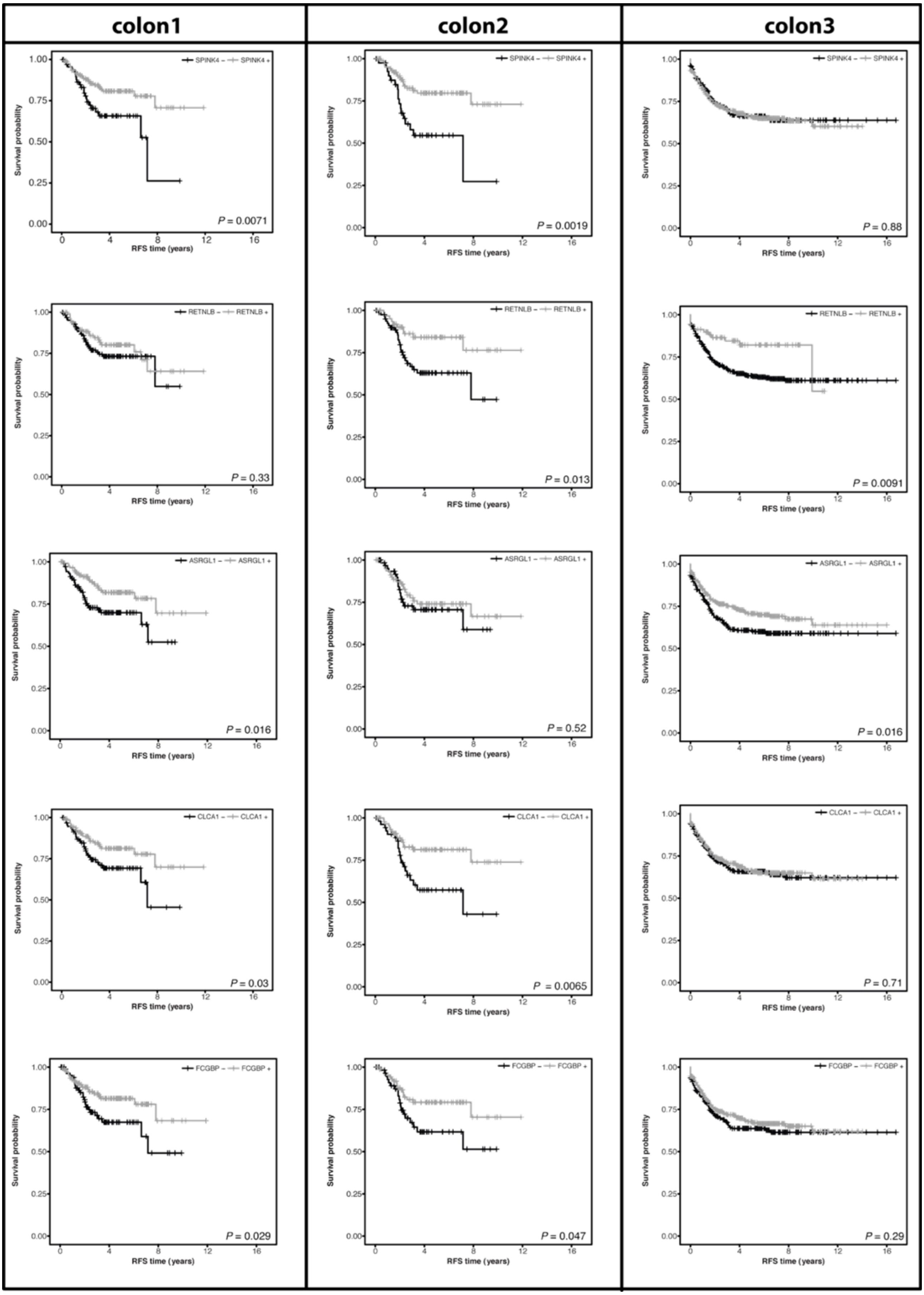

Supplementary Figure 6. Kaplan-Meier curves showing disease-free survival for the three colon cancer data sets (columns). The curves present survival data for the two groups of colon cancer patients based on gene expression level (high or low) of SPINK4, RETNLB, ASRGL1, CLCA1, and FCGBP (rows). The grey curves correspond to patients with high gene expression and the black curves correspond to patients with low gene expression. The *x*-axis shows disease-free survival time in years and the *y*-axis shows the probability of disease-free survival. The log-rank test was used to compare survival rates between the two expression groups (high and low).

Supplementary Figure 7

A

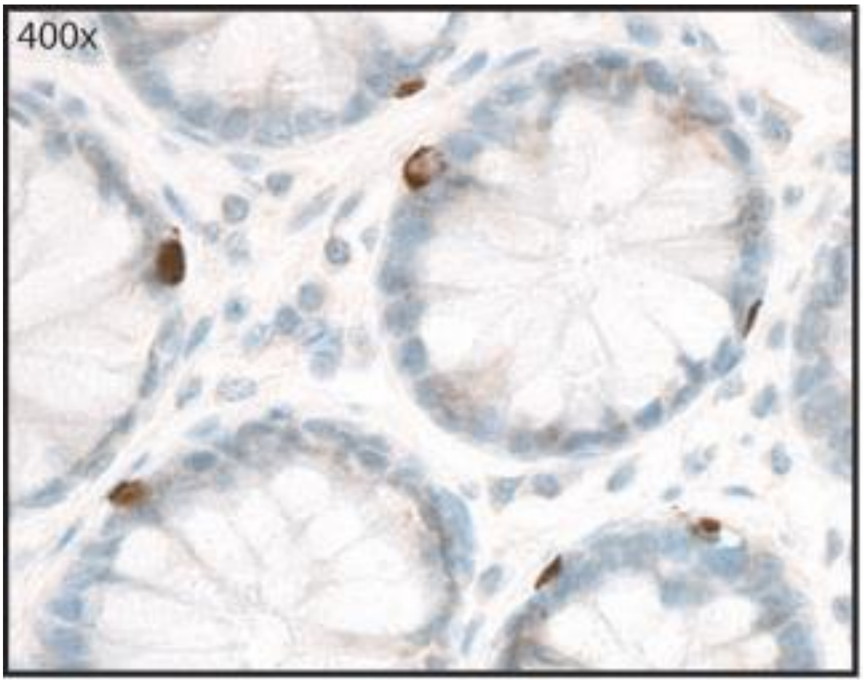

Healthy tissue

B

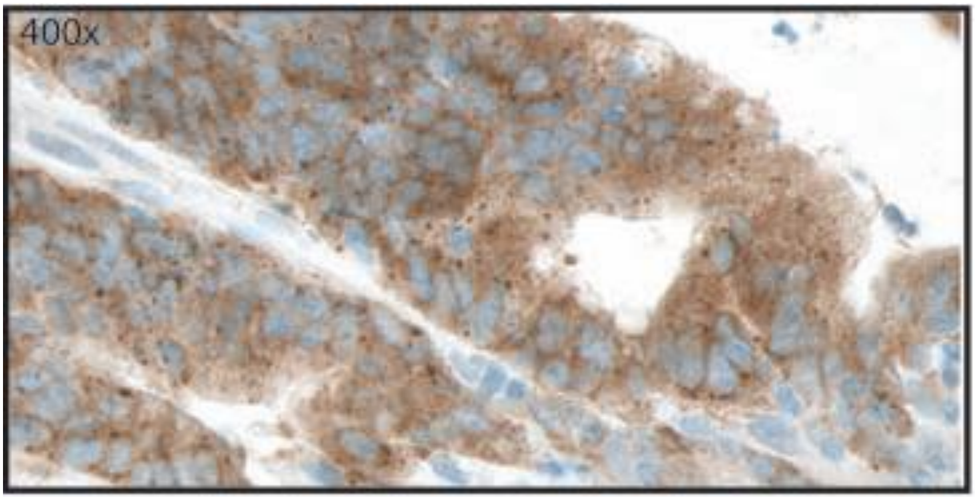

Colorectal adenocarcinoma

Supplementary Figure 7. Formalin-fixed and paraffin-embedded tissue microarray blocks were stained with immunohistochemistry. **(A)** L1TD1 expression analyzed by anti-L1TD1 (Atlas Antibodies, HPA028501) [brown] staining of normal tissue section revealed organized and regulated expression of L1TD1. **(B)** Staining of colorectal adenocarcinoma samples revealed high levels of L1TD1 expression. Representative images were chosen from ten tissue microarray blocks that were stained for L1TD1 expression.
