# Supplementary material for: L1TD1 - a prognostic marker for colon cancer
Source: BMC Cancer. 2019 Jul 23;19:727. doi: 10.1186/s12885-019-5952-2 (PMC6651905; doi:10.1186/s12885-019-5952-2)
Supplement: Supplementary file 2 — Table S1. 311 Interaction partners of L1TD1 were determined using Mass spectrometry and co-immunoprecipitation in our earlier publication (Emani, Närvä, 2015, Stem cell reports). 306 Interaction partners of L1TD1 were identified by performing a Mass spectrometry analysis on co-immunoprecipitated proteins with two different anti-L1TD1 antibodies (recognizing different epitopes on L1TD1). In addition, we included 5 more proteins (NANOG, OCT4 (POU5F1), SOX2, DNMT3B, and TRIM28) that were challenging to detect using Mass spectrometry but the interactions were shown using Immunoprecipitation and Western Blotting. This makes a total of 311 proteins that are referred to in this work as “Interaction partners” of L1TD1. Table S2. Colon cancer samples with a high L1TD1 expression and a concomitant lack of expression of the listed interaction partner were compared to colon cancer samples with a low L1TD1 expression in the three data sets, this table lists the P-values (log-rank test) for these comparisons. P-value less (more significant) than the one obtained by comparing L1TD1 high and low sample groups are highlighted. Table S3. Table lists the 20 genes that had a positive correlation with L1TD1 in the colon cancer data sets. The table lists their UNIPROT ID and UNIRPOT protein name. (PDF 211 kb) [file 12885_2019_5952_MOESM2_ESM.pdf]

SI Table 1

311 Interaction partners of L1TD1 were determined using Mass spectrometry and co-immunoprecipitation in our earlier publication (Emani, Närvä, 2015, Stem cell reports). 306 Interaction partners of L1TD1 were identified by performing a Mass spectrometry analysis on co-immunoprecipitated proteins with two different anti-L1TD1 antibodies (recognizing different epitopes on L1TD1). In addition, we included 5 more proteins (NANOG, OCT4 (POU5F1), SOX2, DNMT3B, and TRIM28) that were challenging to detect using Mass spectrometry but the interactions were shown using Immunoprecipitation and Western Blotting. This makes a total of 311 proteins that are referred to in this work as "Interaction partners" of L1TD1.

#	Interaction Partner name	Entry	Entry name	Protein names	All gene names
1	L1TD1	Q5T7N2	LITD1_HUMAN	LINE-1 type transposase domain-containing protein 1	L1TD1, ECAT11
2	ACTBL2	Q562R1	ACTBL_HUMAN	Beta-actin-like protein 2	ACTBL2
3	ACTC1	P68032	ACTC_HUMAN	Actin, alpha cardiac muscle 1	ACTC1, ACTC
4	ACTN1	P12814	ACTN1_HUMAN	Alpha-actinin-1	ACTN1
5	ACTR2	P61160	ARP2_HUMAN	Actin-related protein 2	ACTR2, ARP2
6	ACTR3	P61158	ARP3_HUMAN	Actin-related protein 3	ACTR3, ARP3
7	ALPL	P05186	PPBT_HUMAN	Alkaline phosphatase, tissue-nonspecific isozyme	ALPL
8	AP2A1	O95782	AP2A1_HUMAN	AP-2 complex subunit alpha-1	AP2A1, ADTAA, CLAPA1
9	AP2A2	O94973	AP2A2_HUMAN	AP-2 complex subunit alpha-2	AP2A2, ADTAB, CLAPA2, HIP9, HYPJ, KIAA0899
10	AP2B1	P63010	AP2B1_HUMAN	AP-2 complex subunit beta	AP2B1, ADTB2, CLAPB1
11	AP2M1	Q96CW1	AP2M1_HUMAN	AP-2 complex subunit mu	AP2M1, CLAPM1, KIAA0109
12	ARF4	P18085	ARF4_HUMAN	ADP-ribosylation factor 4	ARF4, ARF2
13	ARPC1A	Q92747	ARC1A_HUMAN	Actin-related protein 2/3 complex subunit 1A	ARPC1A, SOP2L
14	ARPC1B	O15143	ARC1B_HUMAN	Actin-related protein 2/3 complex subunit 1B	ARPC1B, ARC41
15	ARPC2	O15144	ARPC2_HUMAN	Actin-related protein 2/3 complex subunit 2	ARPC2, ARC34, PRO2446
16	ARPC3	O15145	ARPC3_HUMAN	Actin-related protein 2/3 complex subunit 3	ARPC3, ARC21
17	ATAD3A	Q9NVI7	ATD3A_HUMAN	ATPase family AAA domain-containing protein 3A	ATAD3A
18	ATP5A1	P25705	ATPA_HUMAN	ATP synthase subunit alpha, mitochondrial	ATP5A1, ATP5A, ATP5AL2, ATPM
19	ATP5C1	P36542	ATPG_HUMAN	ATP synthase subunit gamma, mitochondrial	ATP5C1, ATP5C, ATP5CL1
20	ATP6V0D1	P61421	VA0D1_HUMAN	V-type proton ATPase subunit d 1	ATP6V0D1, ATP6D, VPATPD
21	BCLAF1	Q9NYF8	BCLF1_HUMAN	Bcl-2-associated transcription factor 1	BCLAF1, BTF, KIAA0164
22	C22orf28	Q9Y3I0	RTCB_HUMAN	tRNA-splicing ligase RtcB homolog	RTCB C22orf28, HSPC117
23	CALD1	Q05682	CALD1_HUMAN	Caldesmon	CALD1, CAD, CDM
24	CAPZA1	P52907	CAZA1_HUMAN	F-actin-capping protein subunit alpha-1	CAPZA1
25	CAPZB	P47756	CAPZB_HUMAN	F-actin-capping protein subunit beta	CAPZB
26	CCT3	P49368	TCPG_HUMAN	T-complex protein 1 subunit gamma	CCT3, CCTG, TRIC5
27	CCT4	P50991	TCPD_HUMAN	T-complex protein 1 subunit delta	CCT4, CCTD, SRB
28	CCT6A	P40227	TCPZ_HUMAN	T-complex protein 1 subunit zeta	CCT6A, CCT6, CCTZ
29	CCT7	Q99832	TCPH_HUMAN	T-complex protein 1 subunit eta	CCT7, CCTH, NIP7-1
30	CCT8	P50990	TCPQ_HUMAN	T-complex protein 1 subunit theta	CCT8, C21orf112, CCTQ, KIAA0002
31	CDC5L	Q99459	CDC5L_HUMAN	Cell division cycle 5-like protein	CDC5L, KIAA0432, PCDC5RP
32	CDK1	P06493	CDK1_HUMAN	Cyclin-dependent kinase 1	CDK1, CDC2, CDC28A, CDKN1, P34CDC2
33	CETN3	O15182	CETN3_HUMAN	Centrin-3	CETN3, CEN3

34	CGN	Q9P2M7	CING_HUMAN	Cingulin	CGN, KIAA1319
35	CHTOP	Q9Y3Y2	CHTOP_HUMAN	Chromatin target of PRMT1 protein	CHTOP, C1orf77, FOP, HT031, PP7704
36	CKMT1A	P12532	KCRU_HUMAN	Creatine kinase U-type, mitochondrial	CKMT1A, CKMT;, CKMT1B, CKMT
37	CLINT1	Q14677	EPN4_HUMAN	Clathrin interactor 1	CLINT1, ENTH, EPN4, EPNR, KIAA0171
38	CLTA	P09496	CLCA_HUMAN	Clathrin light chain A	CLTA
39	CLTC	Q00610	CLH1_HUMAN	Clathrin heavy chain 1	CLTC, CLH17, CLTCL2, KIAA0034
40	COL18A1	P39060	COIA1_HUMAN	Collagen alpha-1	COL18A1
41	CORO1C	Q9ULV4	COR1C_HUMAN	Coronin-1C	CORO1C, CRN2, CRNN4
42	CSNK1A1	P48729	KC1A_HUMAN	Casein kinase I isoform alpha	CSNK1A1
43	CSNK2A1	P68400	CSK21_HUMAN	Casein kinase II subunit alpha	CSNK2A1, CK2A1
44	CSNK2B	P67870	CSK2B_HUMAN	Casein kinase II subunit beta	CSNK2B, CK2N, G5A
45	CTNNA1	P35221	CTNA1_HUMAN	Catenin alpha-1	CTNNA1
46	CTNNB1	P35222	CTNB1_HUMAN	Catenin beta-1	CTNNB1, CTNNB, OK/SW-cl.35, PRO2286
47	CTNND1	O60716	CTND1_HUMAN	Catenin delta-1	CTNND1, KIAA0384
48	CTTN	Q14247	SRC8_HUMAN	Src substrate cortactin	CTTN, EMS1
49	DAP3	P51398	RT29_HUMAN	28S ribosomal protein S29, mitochondrial	DAP3, MRPS29
50	DARS	P14868	SYDC_HUMAN	Aspartate--tRNA ligase, cytoplasmic	DARS, PIG40
51	DDX17	Q92841	DDX17_HUMAN	Probable ATP-dependent RNA helicase DDX17	DDX17
52	DDX21	Q9NR30	DDX21_HUMAN	Nucleolar RNA helicase 2	DDX21
53	DDX3X	O00571	DDX3X_HUMAN	ATP-dependent RNA helicase DDX3X	DDX3X, DBX, DDX3
54	DDX5	P17844	DDX5_HUMAN	Probable ATP-dependent RNA helicase DDX5	DDX5, G17P1, HELR, HLR1
55	DHX9	Q08211	DHX9_HUMAN	ATP-dependent RNA helicase A	DHX9, DDX9, LKP, NDH2
56	DIMT1L	Q9UNQ2	DIM1_HUMAN	Probable dimethyladenosine transferase	DIMT1 DIMT1L, HUSSY-05
57	DKC1	O60832	DKC1_HUMAN	H/ACA ribonucleoprotein complex subunit 4	DKC1, NOLA4
58	DNAJA2	O60884	DNJA2_HUMAN	DnaJ homolog subfamily A member 2	DNAJA2, CPR3, HIRIP4
59	DNAJA3	Q96EY1	DNJA3_HUMAN	DnaJ homolog subfamily A member 3, mitochondrial	DNAJA3, HCA57, TID1
60	DNMT3B	Q9UBC3	DNM3B_HUMAN	DNA (cytosine-5)-methyltransferase 3B	DNMT3B
61	DPPA4	Q7L190	DPPA4_HUMAN	Developmental pluripotency-associated protein 4	DPPA4
62	DSP	P15924	DESP_HUMAN	Desmoplakin	DSP
63	EBNA1BP2	Q99848	EBP2_HUMAN	Probable rRNA-processing protein EBP2	EBNA1BP2, EBP2
64	EEF1D	P29692	EF1D_HUMAN	Elongation factor 1-delta	EEF1D, EF1D
65	EEF1E1	O43324	MCA3_HUMAN	Eukaryotic translation elongation factor 1 epsilon-1	EEF1E1, AIMP3, P18
66	EEF1G	P26641	EF1G_HUMAN	Elongation factor 1-gamma	EEF1G, EF1G, PRO1608
67	EEF2	P13639	EF2_HUMAN	Elongation factor 2	EEF2, EF2
68	EFTUD2	Q15029	U5S1_HUMAN	116 kDa U5 small nuclear ribonucleoprotein component	EFTUD2, KIAA0031, SNRP116
69	EIF3B	P55884	EIF3B_HUMAN	Eukaryotic translation initiation factor 3 subunit B	EIF3B, EIF3S9
70	EIF3E	P60228	EIF3E_HUMAN	Eukaryotic translation initiation factor 3 subunit E	EIF3E, EIF3S6, INT6
71	EIF3F	O00303	EIF3F_HUMAN	Eukaryotic translation initiation factor 3 subunit F	EIF3F, EIF3S5
72	EIF3H	O15372	EIF3H_HUMAN	Eukaryotic translation initiation factor 3 subunit H	EIF3H, EIF3S3
73	EIF3I	Q13347	EIF3I_HUMAN	Eukaryotic translation initiation factor 3 subunit I	EIF3I, EIF3S2, TRIP1

74	EIF3K	Q9UBQ5	EIF3K_HUMAN	Eukaryotic translation initiation factor 3 subunit K	EIF3K, EIF3S12, ARG134, HSPC029, MSTP001, PTD001
75	EIF3L	Q9Y262	EIF3L_HUMAN	Eukaryotic translation initiation factor 3 subunit L	EIF3L, EIF3EIP, EIF3S6IP, HSPC021, HSPC025, MSTP005
76	EIF3M	Q7L2H7	EIF3M_HUMAN	Eukaryotic translation initiation factor 3 subunit M	EIF3M, HFLB5, PCID1, GA17, PNAS-125
77	EIF4A1	P60842	IF4A1_HUMAN	Eukaryotic initiation factor 4A-I	EIF4A1, DDX2A, EIF4A
78	EIF4A3	P38919	IF4A3_HUMAN	Eukaryotic initiation factor 4A-III	EIF4A3, DDX48, KIAA0111
79	ELAVL1	Q15717	ELAV1_HUMAN	ELAV-like protein 1	ELAVL1, HUR
80	EMD	P50402	EMD_HUMAN	Emerin	EMD, EDMD, STA
81	ENO1	P06733	ENOA_HUMAN	Alpha-enolase	ENO1, ENO1L1, MBPB1, MPB1
82	EXOSC2	Q13868	EXOS2_HUMAN	Exosome complex component RRP4	EXOSC2, RRP4
83	FBL	P22087	FBRL_HUMAN	rRNA 2'-O-methyltransferase fibrillarin	FBL, FIB1, FLRN
84	FLNB	O75369	FLNB_HUMAN	Filamin-B	FLNB, FLN1L, FLN3, TABP, TAP
85	FLOT1	O75955	FLOT1_HUMAN	Flotillin-1	FLOT1
86	FLOT2	Q14254	FLOT2_HUMAN	Flotillin-2	FLOT2, ESA1, M17S1
87	FN1	P02751	FINC_HUMAN	Fibronectin	FN1, FN
88	FSCN1	Q16658	FSCN1_HUMAN	Fascin	FSCN1, FAN1, HSN, SNL
89	FXR1	P51114	FXR1_HUMAN	Fragile X mental retardation syndrome-related protein 1	FXR1
90	G3BP2	Q9UN86	G3BP2_HUMAN	Ras GTPase-activating protein-binding protein 2	G3BP2, KIAA0660
91	GAPDH	P04406	G3P_HUMAN	Glyceraldehyde-3-phosphate dehydrogenase	GAPDH, GAPD, CDABP0047, OK/SW-cl.12
92	GJA1	P17302	CXA1_HUMAN	Gap junction alpha-1 protein	GJA1, GJAL
93	GLG1	Q92896	GSLG1_HUMAN	Golgi apparatus protein 1	GLG1, CFR1, ESL1, MG160
94	GLYR1	Q49A26	GLYR1_HUMAN	Putative oxidoreductase GLYR1	GLYR1, HIBDL, NP60
95	GNAI2	P04899	GNAI2_HUMAN	Guanine nucleotide-binding protein G	GNAI2, GNAI2B
96	GNB1	P62873	GBB1_HUMAN	Guanine nucleotide-binding protein G	GNB1
97	GNB2	P62879	GBB2_HUMAN	Guanine nucleotide-binding protein G	GNB2
98	GNB2L1	P63244	RACK1_HUMAN	Receptor of activated protein C kinase 1	RACK1 GNB2L1, HLC7, PIG21
99	GPC4	O75487	GPC4_HUMAN	Glypican-4	GPC4, UNQ474/PRO937
100	GSN	P06396	GELS_HUMAN	Gelsolin	GSN
101	GTF2I	P78347	GTF2I_HUMAN	General transcription factor II-I	GTF2I, BAP135, WBSCR6
102	H1F0	P07305	H10_HUMAN	Histone H1.0	H1F0, H1FV
103	H1FX	Q92522	H1X_HUMAN	Histone H1x	H1FX
104	HADHA	P40939	ECHA_HUMAN	Trifunctional enzyme subunit alpha, mitochondrial	HADHA, HADH
105	HNRNPA1	P09651	ROA1_HUMAN	Heterogeneous nuclear ribonucleoprotein A1	HNRNPA1, HNRPA1
106	HNRNPA2B1	P22626	ROA2_HUMAN	Heterogeneous nuclear ribonucleoproteins A2/B1	HNRNPA2B1, HNRPA2B1
107	HNRNPC	P07910	HNRPC_HUMAN	Heterogeneous nuclear ribonucleoproteins C1/C2	HNRNPC, HNRPC
108	HNRNPH1	P31943	HNRH1_HUMAN	Heterogeneous nuclear ribonucleoprotein H	HNRNPH1, HNRPH, HNRPH1
109	HNRNPH3	P31942	HNRH3_HUMAN	Heterogeneous nuclear ribonucleoprotein H3	HNRNPH3, HNRPH3
110	HNRNPK	P61978	HNRPK_HUMAN	Heterogeneous nuclear ribonucleoprotein K	HNRNPK, HNRPK
111	HNRNPL	P14866	HNRPL_HUMAN	Heterogeneous nuclear ribonucleoprotein L	HNRNPL, HNRPL, P/OKcl.14
112	HNRNPU	Q00839	HNRPU_HUMAN	Heterogeneous nuclear ribonucleoprotein U	HNRNPU, C1orf199, HNRPU, SAFA, U21.1

113	HP1BP3	Q5SSJ5	HP1B3_HUMAN	Heterochromatin protein 1-binding protein 3	HP1BP3
114	HSP90AA1	P07900	HS90A_HUMAN	Heat shock protein HSP 90-alpha	HSP90AA1, HSP90A, HSPC1, HSPCA
115	HSP90B1	P14625	ENPL_HUMAN	Endoplasmin	HSP90B1, GRP94, TRA1
116	HSPA5	P11021	GRP78_HUMAN	78 kDa glucose-regulated protein	HSPA5, GRP78
117	HSPA8	P11142	HSP7C_HUMAN	Heat shock cognate 71 kDa protein	HSPA8, HSC70, HSP73, HSPA10
118	HSPD1	P10809	CH60_HUMAN	60 kDa heat shock protein, mitochondrial	HSPD1, HSP60
119	IGF2BP1	Q9NZI8	IF2B1_HUMAN	Insulin-like growth factor 2 mRNA-binding protein 1	IGF2BP1, CRDBP, VICKZ1, ZBP1
120	IGF2BP3	O00425	IF2B3_HUMAN	Insulin-like growth factor 2 mRNA-binding protein 3	IGF2BP3, IMP3, KOC1, VICKZ3
121	ILF2	Q12905	ILF2_HUMAN	Interleukin enhancer-binding factor 2	ILF2, NF45, PRO3063
122	JUP	P14923	PLAK_HUMAN	Junction plakoglobin	JUP, CTNNG, DP3
123	KHDRBS1	Q07666	KHDR1_HUMAN	KH domain-containing, RNA-binding, signal transduction-associated protein 1	KHDRBS1, SAM68
124	KHDRBS3	O75525	KHDR3_HUMAN	KH domain-containing, RNA-binding, signal transduction-associated protein 3	KHDRBS3, SALP, SLM2
125	KPNA2	P52292	IMA1_HUMAN	Importin subunit alpha-1	KPNA2, RCH1, SRP1
126	KRT7	P08729	K2C7_HUMAN	Keratin, type II cytoskeletal 7	KRT7, SCL
127	LAD1	O00515	LAD1_HUMAN	Ladinin-1	LAD1, LAD
128	LAMA1	P25391	LAMA1_HUMAN	Laminin subunit alpha-1	LAMA1, LAMA
129	LAMB1	P07942	LAMB1_HUMAN	Laminin subunit beta-1	LAMB1
130	LAMC1	P11047	LAMC1_HUMAN	Laminin subunit gamma-1	LAMC1, LAMB2
131	LDHB	P07195	LDHB_HUMAN	L-lactate dehydrogenase B chain	LDHB
132	LIN28A	Q9H9Z2	LN28A_HUMAN	Protein lin-28 homolog A	LIN28A, CSDD1, LIN28, ZCCHC1
133	LRPPRC	P42704	LPPRC_HUMAN	Leucine-rich PPR motif-containing protein, mitochondrial	LRPPRC, LRP130
134	LYN	P07948	LYN_HUMAN	Tyrosine-protein kinase Lyn	LYN, JTK8
135	MATR3	P43243	MATR3_HUMAN	Matrin-3	MATR3, KIAA0723
136	MCCC2	Q9HCC0	MCCB_HUMAN	Methylcrotonoyl-CoA carboxylase beta chain, mitochondrial	MCCC2, MCCB
137	MCM5	P33992	MCM5_HUMAN	DNA replication licensing factor MCM5	MCM5, CDC46
138	MCM7	P33993	MCM7_HUMAN	DNA replication licensing factor MCM7	MCM7, CDC47, MCM2
139	MRPL13	Q9BYD1	RM13_HUMAN	39S ribosomal protein L13, mitochondrial	MRPL13
140	MRPL18	Q9H0U6	RM18_HUMAN	39S ribosomal protein L18, mitochondrial	MRPL18, HSPC071
141	MRPL39	Q9NYK5	RM39_HUMAN	39S ribosomal protein L39, mitochondrial	MRPL39, C21orf92, MRPL5, RPML5, MSTP003, PRED22
142	MRPL45	Q9BRJ2	RM45_HUMAN	39S ribosomal protein L45, mitochondrial	MRPL45
143	MRPS10	P82664	RT10_HUMAN	28S ribosomal protein S10, mitochondrial	MRPS10, MSTP040
144	MRPS15	P82914	RT15_HUMAN	28S ribosomal protein S15, mitochondrial	MRPS15, RPMS15, DC37
145	MRPS17	Q9Y2R5	RT17_HUMAN	28S ribosomal protein S17, mitochondrial	MRPS17, RPMS17, HSPC011
146	MRPS18B	Q9Y676	RT18B_HUMAN	28S ribosomal protein S18b, mitochondrial	MRPS18B, C6orf14, HSPC183, PTD017
147	MRPS22	P82650	RT22_HUMAN	28S ribosomal protein S22, mitochondrial	MRPS22, C3orf5, RPMS22, GK002
148	MRPS23	Q9Y3D9	RT23_HUMAN	28S ribosomal protein S23, mitochondrial	MRPS23, CGI-138, HSPC329
149	MRPS25	P82663	RT25_HUMAN	28S ribosomal protein S25, mitochondrial	MRPS25, RPMS25
150	MRPS26	Q9BYN8	RT26_HUMAN	28S ribosomal protein S26, mitochondrial	MRPS26, C20orf193, RPMS13
151	MRPS27	Q92552	RT27_HUMAN	28S ribosomal protein S27, mitochondrial	MRPS27, KIAA0264
152	MRPS28	Q9Y2Q9	RT28_HUMAN	28S ribosomal protein S28, mitochondrial	MRPS28, MRPS35, HSPC007
153	MRPS31	Q92665	RT31_HUMAN	28S ribosomal protein S31, mitochondrial	MRPS31, IMOGN38
154	MRPS35	P82673	RT35_HUMAN	28S ribosomal protein S35, mitochondrial	MRPS35, MRPS28, HDCMD11P, MDS023, PSEC0213

155	MRPS7	Q9Y2R9	RT07_HUMAN	28S ribosomal protein S7, mitochondrial	MRPS7
156	MRPS9	P82933	RT09_HUMAN	28S ribosomal protein S9, mitochondrial	MRPS9, RPMS9
157	MYBBP1A	Q9BQG0	MBB1A_HUMAN	Myb-binding protein 1A	MYBBP1A, P160
158	MYH10	P35580	MYH10_HUMAN	Myosin-10	MYH10
159	MYL12B	O14950	ML12B_HUMAN	Myosin regulatory light chain 12B	MYL12B, MRLC2, MYLC2B
160	MYL6	P60660	MYL6_HUMAN	Myosin light polypeptide 6	MYL6
161	MYO18A	Q92614	MY18A_HUMAN	Unconventional myosin-XVIIIa	MYO18A, CD245, KIAA0216, MYSPDZ
162	MYO1B	O43795	MYO1B_HUMAN	Unconventional myosin-Ib	MYO1B
163	MYO1C	O00159	MYO1C_HUMAN	Unconventional myosin-Ic	MYO1C
164	MYO1D	O94832	MYO1D_HUMAN	Unconventional myosin-Id	MYO1D, KIAA0727
165	NANOG	Q9H9S0	NANOG_HUMAN	Homeobox protein NANOG	NANOG
166	NAP1L1	P55209	NP1L1_HUMAN	Nucleosome assembly protein 1-like 1	NAP1L1, NRP
167	NCL	P19338	NUCL_HUMAN	Nucleolin	NCL
168	NID1	P14543	NID1_HUMAN	Nidogen-1	NID1, NID
169	NOLC1	Q14978	NOLC1_HUMAN	Nucleolar and coiled-body phosphoprotein 1	NOLC1, KIAA0035, NS5ATP13
170	NOP56	O00567	NOP56_HUMAN	Nucleolar protein 56	NOP56, NOL5A
171	NUDT21	O43809	CPSF5_HUMAN	Cleavage and polyadenylation specificity factor subunit 5	NUDT21, CFIM25, CPSF25, CPSF5
172	P4HB	P07237	PDIA1_HUMAN	Protein disulfide-isomerase	P4HB, ERBA2L, PDI, PDIA1, PO4DB
173	PABPC1	P11940	PABP1_HUMAN	Polyadenylate-binding protein 1	PABPC1, PAB1, PABP1, PABPC2
174	PARP1	P09874	PARP1_HUMAN	Poly [ADP-ribose] polymerase 1	PARP1, ADPRT, PPOL
175	PDIA6	Q15084	PDIA6_HUMAN	Protein disulfide-isomerase A6	PDIA6, ERP5, P5, TXNDC7
176	PFN1	P07737	PROF1_HUMAN	Profilin-1	PFN1
177	PGAM5	Q96HS1	PGAM5_HUMAN	Serine/threonine-protein phosphatase PGAM5, mitochondrial	PGAM5
178	PHB	P35232	PHB_HUMAN	Prohibitin	PHB
179	PHB2	Q99623	PHB2_HUMAN	Prohibitin-2	PHB2, BAP, REA
180	PHGDH	O43175	SERA_HUMAN	D-3-phosphoglycerate dehydrogenase	PHGDH, PGDH3
181	PICALM	Q13492	PICAL_HUMAN	Phosphatidylinositol-binding clathrin assembly protein	PICALM, CALM
182	PKP2	Q99959	PKP2_HUMAN	Plakophilin-2	PKP2
183	PLEC	Q15149	PLEC_HUMAN	Plectin	PLEC, PLEC1
184	PLRG1	O43660	PLRG1_HUMAN	Pleiotropic regulator 1	PLRG1
185	POLDIP3	Q9BY77	PDIP3_HUMAN	Polymerase delta-interacting protein 3	POLDIP3, KIAA1649, PDIP46
186	OCT4	Q01860	PO5F1_HUMAN	POU domain, class 5, transcription factor 1	POU5F1, OCT3,, OCT4,, OTF3
187	PPIA	P62937	PPIA_HUMAN	Peptidyl-prolyl cis-trans isomerase A	PPIA, CYPA
188	PPIB	P23284	PPIB_HUMAN	Peptidyl-prolyl cis-trans isomerase B	PPIB, CYPB
189	PPIH	O43447	PPIH_HUMAN	Peptidyl-prolyl cis-trans isomerase H	PPIH, CYP20, CYPH
190	PPP1CA	P62136	PP1A_HUMAN	Serine/threonine-protein phosphatase PP1-alpha catalytic subunit	PPP1CA, PPP1A
191	PPP1CB	P62140	PP1B_HUMAN	Serine/threonine-protein phosphatase PP1-beta catalytic subunit	PPP1CB
192	PPP1R12A	O14974	MYPT1_HUMAN	Protein phosphatase 1 regulatory subunit 12A	PPP1R12A, MBS, MYPT1
193	PRKDC	P78527	PRKDC_HUMAN	DNA-dependent protein kinase catalytic subunit	PRKDC, HYRC, HYRC1
194	PRPF19	Q9UMS4	PRP19_HUMAN	Pre-mRNA-processing factor 19	PRPF19, NMP200, PRP19, SNEV
195	PRPF6	O94906	PRP6_HUMAN	Pre-mRNA-processing factor 6	PRPF6, C20orf14
196	PRPF8	Q6P2Q9	PRP8_HUMAN	Pre-mRNA-processing-splicing factor 8	PRPF8, PRPC8
197	PSMD1	Q99460	PSMD1_HUMAN	26S proteasome non-ATPase regulatory subunit 1	PSMD1

198	PSMD11	O00231	PSD11_HUMAN	26S proteasome non-ATPase regulatory subunit 11	PSMD11
199	PSMD14	O00487	PSDE_HUMAN	26S proteasome non-ATPase regulatory subunit 14	PSMD14, POH1
200	PSMD2	Q13200	PSMD2_HUMAN	26S proteasome non-ATPase regulatory subunit 2	PSMD2, TRAP2
201	PSMD7	P51665	PSMD7_HUMAN	26S proteasome non-ATPase regulatory subunit 7	PSMD7, MOV34L
202	PSMD8	P48556	PSMD8_HUMAN	26S proteasome non-ATPase regulatory subunit 8	PSMD8
203	PTCD3	Q96EY7	PTCD3_HUMAN	Pentatricopeptide repeat domain-containing protein 3, mitochondrial	PTCD3, MRPS39, TRG15
204	PYCR2	Q96C36	P5CR2_HUMAN	Pyrroline-5-carboxylate reductase 2	PYCR2
205	QPCTL	Q9NXS2	QPCTL_HUMAN	Glutaminyl-peptide cyclotransferase-like protein	QPCTL
206	RAE1	P78406	RAE1L_HUMAN	mRNA export factor	RAE1, MRNP41
207	RAI14	Q9P0K7	RAI14_HUMAN	Ankycorbin	RAI14, KIAA1334, NORPEG
208	RALY	Q9UKM9	RALY_HUMAN	RNA-binding protein Raly	RALY, HNRPCL2, P542
209	RAN	P62826	RAN_HUMAN	GTP-binding nuclear protein Ran	RAN, ARA24, OK/SW-cl.81
210	RBBP4	Q09028	RBBP4_HUMAN	Histone-binding protein RBBP4	RBBP4, RBAP48
211	RBMX	P38159	RBMX_HUMAN	RNA-binding motif protein, X chromosome	RBMX, HNRPG, RBMXP1
212	RCL1	Q9Y2P8	RCL1_HUMAN	RNA 3'-terminal phosphate cyclase-like protein	RCL1, RNAC, RPC2, RPCL1, RTC2, HSPC338
213	RCN2	Q14257	RCN2_HUMAN	Reticulocalbin-2	RCN2, ERC55
214	REPS1	Q96D71	REPS1_HUMAN	RalBP1-associated Eps domain-containing protein 1	REPS1
215	RFC4	P35249	RFC4_HUMAN	Replication factor C subunit 4	RFC4
216	RFC5	P40937	RFC5_HUMAN	Replication factor C subunit 5	RFC5
217	RPF2	Q9H7B2	RPF2_HUMAN	Ribosome production factor 2 homolog	RPF2, BXDC1
218	RPL10A	P62906	RL10A_HUMAN	60S ribosomal protein L10a	RPL10A, NEDD6
219	RPL13A	P40429	RL13A_HUMAN	60S ribosomal protein L13a	RPL13A
220	RPL14	P50914	RL14_HUMAN	60S ribosomal protein L14	RPL14
221	RPL15	P61313	RL15_HUMAN	60S ribosomal protein L15	RPL15, EC45, TCBAP0781
222	RPL17	P18621	RL17_HUMAN	60S ribosomal protein L17	RPL17
223	RPL18	Q07020	RL18_HUMAN	60S ribosomal protein L18	RPL18
224	RPL18A	Q02543	RL18A_HUMAN	60S ribosomal protein L18a	RPL18A
225	RPL19	P84098	RL19_HUMAN	60S ribosomal protein L19	RPL19
226	RPL21	P46778	RL21_HUMAN	60S ribosomal protein L21	RPL21
227	RPL22	P35268	RL22_HUMAN	60S ribosomal protein L22	RPL22
228	RPL23	P62829	RL23_HUMAN	60S ribosomal protein L23	RPL23
229	RPL23A	P62750	RL23A_HUMAN	60S ribosomal protein L23a	RPL23A
230	RPL24	P83731	RL24_HUMAN	60S ribosomal protein L24	RPL24
231	RPL27A	P46776	RL27A_HUMAN	60S ribosomal protein L27a	RPL27A
232	RPL28	P46779	RL28_HUMAN	60S ribosomal protein L28	RPL28
233	RPL3	P39023	RL3_HUMAN	60S ribosomal protein L3	RPL3, OK/SW-cl.32
234	RPL32	P62910	RL32_HUMAN	60S ribosomal protein L32	RPL32, PP9932
235	RPL34	P49207	RL34_HUMAN	60S ribosomal protein L34	RPL34
236	RPL36AL	Q969Q0	RL36L_HUMAN	60S ribosomal protein L36a-like	RPL36AL
237	RPL4	P36578	RL4_HUMAN	60S ribosomal protein L4	RPL4, RPL1
238	RPL5	P46777	RL5_HUMAN	60S ribosomal protein L5	RPL5, MSTP030
239	RPL6	Q02878	RL6_HUMAN	60S ribosomal protein L6	RPL6, TXREB1
240	RPL7	P18124	RL7_HUMAN	60S ribosomal protein L7	RPL7
241	RPL7A	P62424	RL7A_HUMAN	60S ribosomal protein L7a	RPL7A, SURF-3, SURF3
242	RPL8	P62917	RL8_HUMAN	60S ribosomal protein L8	RPL8
243	RPLP0	P05388	RLA0_HUMAN	60S acidic ribosomal protein P0	RPLP0
244	RPN1	P04843	RPN1_HUMAN	Dolichyl-diphosphooligosaccharide--protein glycosyltransferase subunit 1	RPN1

245	RPS10	P46783	RS10_HUMAN	40S ribosomal protein S10	RPS10
246	RPS14	P62263	RS14_HUMAN	40S ribosomal protein S14	RPS14, PRO2640
247	RPS16	P62249	RS16_HUMAN	40S ribosomal protein S16	RPS16
248	RPS2	P15880	RS2_HUMAN	40S ribosomal protein S2	RPS2, RPS4
249	RPS23	P62266	RS23_HUMAN	40S ribosomal protein S23	RPS23
250	RPS24	P62847	RS24_HUMAN	40S ribosomal protein S24	RPS24
251	RPS26	P62854	RS26_HUMAN	40S ribosomal protein S26	RPS26
252	RPS5	P46782	RS5_HUMAN	40S ribosomal protein S5	RPS5
253	RPS6	P62753	RS6_HUMAN	40S ribosomal protein S6	RPS6, OK/SW-cl.2
254	RPS7	P62081	RS7_HUMAN	40S ribosomal protein S7	RPS7
255	RPS8	P62241	RS8_HUMAN	40S ribosomal protein S8	RPS8, OK/SW-cl.83
256	RPS9	P46781	RS9_HUMAN	40S ribosomal protein S9	RPS9
257	RPSA	P08865	RSSA_HUMAN	40S ribosomal protein SA	RPSA, LAMBR, LAMR1
258	RRS1	Q15050	RRS1_HUMAN	Ribosome biogenesis regulatory protein homolog	RRS1, KIAA0112, RRR
259	RUVBL1	Q9Y265	RUVB1_HUMAN	RuvB-like 1	RUVBL1, INO80H, NMP238, TIP49, TIP49A
260	RUVBL2	Q9Y230	RUVB2_HUMAN	RuvB-like 2	RUVBL2, INO80J, TIP48, TIP49B, CGI-46
261	SAP18	O00422	SAP18_HUMAN	Histone deacetylase complex subunit SAP18	SAP18, GIG38
262	SEC13	P55735	SEC13_HUMAN	Protein SEC13 homolog	SEC13, D3S1231E, SEC13L1, SEC13R
263	SERPINH1	P50454	SERPH_HUMAN	Serpin H1	SERPINH1, CBP1, CBP2, HSP47, SERPINH2, PIG14
264	SF3A1	Q15459	SF3A1_HUMAN	Splicing factor 3A subunit 1	SF3A1, SAP114
265	SF3B1	O75533	SF3B1_HUMAN	Splicing factor 3B subunit 1	SF3B1, SAP155
266	SFPQ	P23246	SFPQ_HUMAN	Splicing factor, proline- and glutamine-rich	SFPQ, PSF
267	SHMT2	P34897	GLYM_HUMAN	Serine hydroxymethyltransferase, mitochondrial	SHMT2
268	SLC25A1	P53007	TXTP_HUMAN	Tricarboxylate transport protein, mitochondrial	SLC25A1, SLC20A3
269	SLC25A3	Q00325	MPCP_HUMAN	Phosphate carrier protein, mitochondrial	SLC25A3, PHC, OK/SW-cl.48
270	SLC25A5	P05141	ADT2_HUMAN	ADP/ATP translocase 2	SLC25A5, ANT2
271	SMPDL3B	Q92485	ASM3B_HUMAN	Acid sphingomyelinase-like phosphodiesterase 3b	SMPDL3B, ASML3B, ASMLPD
272	SMU1	Q2TAY7	SMU1_HUMAN	WD40 repeat-containing protein SMU1	SMU1
273	SNRNP200	O75643	U520_HUMAN	U5 small nuclear ribonucleoprotein 200 kDa helicase	SNRNP200, ASCC3L1, HELIC2, KIAA0788
274	SNRNP70	P08621	RU17_HUMAN	U1 small nuclear ribonucleoprotein 70 kDa	SNRNP70, RNPU1Z, RPU1, SNRP70, U1AP1
275	SNRPB	P14678	RSMB_HUMAN	Small nuclear ribonucleoprotein-associated proteins B and B'	SNRPB, COD, SNRPB1
276	SOX2	P48431	SOX2_HUMAN	Transcription factor SOX-2	SOX2
277	SRSF1	Q07955	SRSF1_HUMAN	Serine/arginine-rich splicing factor 1	SRSF1, ASF, SF2, SF2P33, SFRS1, OK/SW-cl.3
278	SRSF3	P84103	SRSF3_HUMAN	Serine/arginine-rich splicing factor 3	SRSF3, SFRS3, SRP20
279	SRSF7	Q16629	SRSF7_HUMAN	Serine/arginine-rich splicing factor 7	SRSF7, SFRS7
280	SRSF9	Q13242	SRSF9_HUMAN	Serine/arginine-rich splicing factor 9	SRSF9, SFRS9, SRP30C
281	SSRP1	Q08945	SSRP1_HUMAN	FACT complex subunit SSRP1	SSRP1, FACT80
282	TCP1	P17987	TCPA_HUMAN	T-complex protein 1 subunit alpha	TCP1, CCT1, CCTA
283	TEX10	Q9NXF1	TEX10_HUMAN	Testis-expressed protein 10	TEX10, L18, Nbla10363
284	THOC6	Q86W42	THOC6_HUMAN	THO complex subunit 6 homolog	THOC6, WDR58, PSEC0006
285	THRAP3	Q9Y2W1	TR150_HUMAN	Thyroid hormone receptor-associated protein 3	THRAP3, BCLAF2, TRAP150
286	THY1	P04216	THY1_HUMAN	Thy-1 membrane glycoprotein	THY1
287	TJP1	Q07157	ZO1_HUMAN	Tight junction protein ZO-1	TJP1, ZO1

288	TJP2	Q9UDY2	ZO2_HUMAN	Tight junction protein ZO-2	TJP2, X104, ZO2
289	TMOD3	Q9NYL9	TMOD3_HUMAN	Tropomodulin-3	TMOD3
290	TOP2A	P11388	TOP2A_HUMAN	DNA topoisomerase 2-alpha	TOP2A, TOP2
291	TPM1	P09493	TPM1_HUMAN	Tropomyosin alpha-1 chain	TPM1, C15orf13, TMSA
292	TRA2B	P62995	TRA2B_HUMAN	Transformer-2 protein homolog beta	TRA2B, SFRS10
293	TRIM28	Q13263	TIF1B_HUMAN	Transcription intermediary factor 1-beta	TRIM28, KAP1, RNF96, TIF1B
294	TRIM71	Q2Q1W2	LIN41_HUMAN	E3 ubiquitin-protein ligase TRIM71	TRIM71, LIN41
295	TUBB	P07437	TBB5_HUMAN	Tubulin beta chain	TUBB, TUBB5, OK/SW-cl.56
296	TUFM	P49411	EFTU_HUMAN	Elongation factor Tu, mitochondrial	TUFM
297	TWF1	Q12792	TWF1_HUMAN	Twinfilin-1	TWF1, PTK9
298	U2AF1	Q01081	U2AF1_HUMAN	Splicing factor U2AF 35 kDa subunit	U2AF1, U2AF35, U2AFBP, FP793
299	UGP2	Q16851	UGPA_HUMAN	UTP--glucose-1-phosphate uridylyltransferase	UGP2, UGP1
300	UTP15	Q8TED0	UTP15_HUMAN	U3 small nucleolar RNA-associated protein 15 homolog	UTP15
301	VCP	P55072	TERA_HUMAN	Transitional endoplasmic reticulum ATPase	VCP
302	VIL1	P09327	VILI_HUMAN	Villin-1	VIL1, VIL
303	WDR18	Q9BV38	WDR18_HUMAN	WD repeat-containing protein 18	WDR18
304	WDR61	Q9GZS3	WDR61_HUMAN	WD repeat-containing protein 61	WDR61
305	WDR82	Q6UXN9	WDR82_HUMAN	WD repeat-containing protein 82	WDR82, TMEM113, WDR82A, UNQ9342/PRO34047
306	XRCC1	P18887	XRCC1_HUMAN	DNA repair protein XRCC1	XRCC1
307	XRCC5	P13010	XRCC5_HUMAN	X-ray repair cross-complementing protein 5	XRCC5, G22P2
308	XRCC6	P12956	XRCC6_HUMAN	X-ray repair cross-complementing protein 6	XRCC6, G22P1
309	YWHAE	P62258	1433E_HUMAN	14-3-3 protein epsilon	YWHAE
310	YWHAG	P61981	1433G_HUMAN	14-3-3 protein gamma	YWHAG
311	YWHAZ	P63104	1433Z_HUMAN	14-3-3 protein zeta/delta	YWHAZ

SI Table 2 – Colon cancer samples with a high L1TD1 expression and a concomitant lack of expression of the listed interaction partner were compared to colon cancer samples with a low L1TD1 expression in the three data sets, this table lists the *P*-values (log-rank test) for these comparisons. P-value less (more significant) than the one obtained by comparing L1TD1 high and low sample groups are highlighted.

		Colon 1		Colon 2		Colon 3	
**Rank**	**Gene Name**	**L1TD1+ vs L1TD1-**	**L1TD1+ & Gene- vs L1TD1-**	**L1TD1+ vs L1TD1-**	**L1TD1+ & Gene- vs L1TD1-**	**L1TD1+ vs L1TD1-**	**L1TD1+ & Gene- vs L1TD1-**
1	**OCT4**		0.01208		0.00896		0.01208
2	**TRIM71**		0.01109		0.01016		0.03896
3	**DPPA4**		0.00973		0.00852		0.01771
4	**DNMT3B**		0.01037		0.00905		0.01587
5	**LRPPRC**		0.07873		-		0.07438
6	**MRPS17**		-		-		-
7	**PARP1**		-		-		0.95881
8	**RPF2**		-		-		-
9	**HSP90AA1**		-		-		-
10	**IGF2BP1**	**0.00973**	0.00973	**0.00852**	0.00852	**0.01861**	0.01771
11	**DNAJA2**		-		-		0.35284
12	**NANOG**		0.00973		0.00852		0.01771
13	**ALPL**		0.00973		0.00852		0.01801
14	**EIF3B**		-		-		-
15	**NCL**		0.00973		0.00852		-
16	**LIN28A**		0.00973		0.00852		0.01771
17	**NOLC1**		0.32871		0.01016		0.02896
18	**CCT8**		0.00973		-		-
19	**RRS1**		-		-		-
20	**SFPQ**		-		-		-

SI Table 3 – Table lists the 20 genes that had a positive correlation with L1TD1 in the colon cancer data sets. The table lists their UNIPROT ID and UNIRPOT protein name.

GENE NAME	UNIOPROT ID (HUMAN)	UNIPROT ENTRY NAME	UNIPROT PROTEIN NAME
RETNLB	Q9BQ08	RETNB_HUMAN	Resistin-like beta
CLCA1	A8K7I4	CLCA1_HUMAN	Calcium-activated chloride channel regulator 1
HEPACAM2	A8MVW5	HECA2_HUMAN	HEPACAM family member 2
FOXA3	P55318	FOXA3_HUMAN	Hepatocyte nuclear factor 3-gamma
FCGBP	Q9Y6R7	FCGBP_HUMAN	IgGFc-binding protein
ST6GALNAC1	Q9NSC7	SIA7A_HUMAN	Alpha-N-acetylgalactosaminide alpha-2,6-sialyltransferase 1
SPINK4	O60575	ISK4_HUMAN	Serine protease inhibitor Kazal-type 4
KIAA1324	Q6UXG2	K1324_HUMAN	UPF0577 protein KIAA1324
KLF4	O43474	KLF4_HUMAN	Krueppel-like factor 4
GMDS	O60547	GMDS_HUMAN	GDP-mannose 4,6 dehydratase
SLITRK6	Q9H5Y7	SLIK6_HUMAN	SLIT and NTRK-like protein 6
SERPINA1	P01009	A1AT_HUMAN	Alpha-1-antitrypsin
LINC00261	-	-	*Long Intergenic Non-Protein Coding RNA 261*
ITLN1	Q8WWA0	ITLN1_HUMAN	Intelectin-1
MUC2	Q02817	MUC2_HUMAN	Mucin-2
DEFA5	Q01523	DEF5_HUMAN	Defensin-5
ASRGL1	Q7L266	ASGL1_HUMAN	Isoaspartyl peptidase/L-asparaginase
SLC27A2	O14975	S27A2_HUMAN	Very long-chain acyl-CoA synthetase
RNF186	Q9NXI6	RN186_HUMAN	RING finger protein 186
PCCA	P05165	PCCA_HUMAN	Propionyl-CoA carboxylase alpha chain
